# Not attackable or not crackable—How pre‐ and post‐attack defenses with different competition costs affect prey coexistence and population dynamics

**DOI:** 10.1002/ece3.4145

**Published:** 2018-06-11

**Authors:** Elias Ehrlich, Ursula Gaedke

**Affiliations:** ^1^ Department of Ecology and Ecosystem Modelling University of Potsdam Potsdam Germany

**Keywords:** coexistence, competition–defense trade‐off, defense against predation, functional response, indirect facilitation, predator–prey cycles

## Abstract

It is well‐known that prey species often face trade‐offs between defense against predation and competitiveness, enabling predator‐mediated coexistence. However, we lack an understanding of how the large variety of different defense traits with different competition costs affects coexistence and population dynamics. Our study focusses on two general defense mechanisms, that is, pre‐attack (e.g., camouflage) and post‐attack defenses (e.g., weaponry) that act at different phases of the predator—prey interaction. We consider a food web model with one predator, two prey types and one resource. One prey type is undefended, while the other one is pre‐ or post‐attack defended paying costs either by a higher half‐saturation constant for resource uptake or a lower maximum growth rate. We show that post‐attack defenses promote prey coexistence and stabilize the population dynamics more strongly than pre‐attack defenses by interfering with the predator's functional response: Because the predator spends time handling “noncrackable” prey, the undefended prey is indirectly facilitated. A high half‐saturation constant as defense costs promotes coexistence more and stabilizes the dynamics less than a low maximum growth rate. The former imposes high costs at low resource concentrations but allows for temporally high growth rates at predator‐induced resource peaks preventing the extinction of the defended prey. We evaluate the effects of the different defense mechanisms and costs on coexistence under different enrichment levels in order to vary the importance of bottom‐up and top‐down control of the prey community.

## 1 INTRODUCTION

Predation and competition for resources represent two major factors determining the survival of species at low and intermediate trophic levels (Sih, Crowley, McPeek, Petranka, & Strohmeier, 1985). Hence, there is strong selection for increasing defense against predation and competitiveness. However, species optimizing one functional trait commonly have to pay costs regarding other traits due to physiological, energetic, and genetic constraints (Stearns, 1989). Trade‐offs between defense and competitiveness have been frequently found in nature and may explain the high diversity of strategies along the gradient of being defended or highly competitive (Agrawal, 1998; Coley, Bryant, & Chapin, 1985; Hillebrand, Worm, & Lotze, 2000). Predation may enable coexistence of competing species facing a trade‐off between defense and competitiveness. This mechanism is known as keystone predation (Leibold, 1996; Menge, Berlow, Blanchette, Navarrete, & Yamada, 1994; Paine, 1966) or analogously as killing the winner in a microbial context (Thingstad, 2000; Winter, Bouvier, Weinbauer, & Thingstad, 2010): A competitive superior species is suppressed by predation promoting the inferior but defended competitor which allows them to coexist. Several studies highlighted the importance of this predator‐mediated coexistence in experimental and natural communities (e.g., Ciros‐Pérez, Carmona, Lapesa, & Serra, 2004; Fauth & Resetarits, 1991; McPeek, 1998).

Species evolved a variety of defense strategies to reduce their predation risk ranging from camouflage, apparent dead, mimicry, aposematism, warning calls, weaponry, chemical defense to escape behavior (Endler, 1991; Lima & Dill, 1990). These defense mechanisms interact in different ways with the predator. Some of them even hamper the predator, for example, chemical defenses, while others do not, for example, camouflage, which may have strong implications for the occurrence of predator‐mediated coexistence. Furthermore, the type of defense costs regarding competitiveness (resource uptake affinity or growth rate) plays an important role for coexistence. Theory already showed that predator‐mediated coexistence crucially depends on the environmental conditions (Chase et al., 2002), for example, the enrichment level (Genkai‐Kato & Yamamura, 1999; Leibold, 1996; Proulx & Mazumder, 1998), the prey switching behavior of the predator (Abrams & Matsuda, 1993; Fryxell & Lundberg, 1994; Murdoch, 1969), the magnitude of the trade‐off between defense and competitiveness (Abrams, 1999; Kasada, Yamamichi, & Yoshida, 2014; Tirok & Gaedke, 2010), and the difference in both the defense level and the competitiveness between the prey types (Becks, Ellner, Jones, & Hairston, 2010; Ehrlich, Becks, & Gaedke, 2017; Jones & Ellner, 2007). However, the role of different defense mechanisms and competition costs in prey communities remains unclear but holds promise to be decisive for their coexistence and the occurring population dynamics. Here, we want to evaluate explicitly the effects of different defense mechanisms and compare the influence of different defense costs in terms of competitiveness on prey coexistence and population dynamics.

Following Bateman, Vos, and Anholt (2014), we distinguish between two general types of defense mechanisms: pre‐attack and post‐attack defenses that act at different phases of the predation sequence, that is, prey encounter, detection, attack, capture, manipulation, consumption, and digestion. A pre‐attack defense implies that the predator does not attack the prey, for example, because it senses the defense, the prey is camouflaged or avoids habitats where predators occur. A post‐attack defense means that the prey is attacked but survives, for example, due to weaponry, escape behavior or robustness. In contrast to pre‐attack defenses, the predator invests time and energy to handle prey with a post‐attack defense which reduces its potential to consume another undefended prey (see Table 1). Hence, a post‐attack defended prey interferes with the functional response of the predator for edible prey which may result in a lower top‐down control of the total prey community, while pre‐attack defended prey does not. Thus, we expect different coexistence patterns and population dynamics in dependence of the defense mechanism.

**Table 1:** Comparison of the predator's handling time spent per prey individual and the resulting energy gain of the predator for an undefended prey (a) and for prey types with three different defense mechanisms (b–d). *T*_a_ represents the time needed for attacking and capturing a prey individual and *T*_m_ is the manipulation and digestion time spent after capturing the prey

Defense mechanism		Handling time	Energy gain	Examples
a) Undefended (*p*_2_ = 1, *q*_2_ = 1)	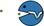	*T*_a_ + *T*_m_	Yes	–
b) Pre‐attack defense (*p*_1_ = 0, *q*_1_ = 1)	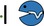	0	No	Camouflage, mimicry, aposematism, apparent dead
c) Post‐attack defense (*p*_1_ = 1, *q*_1_ = 0)	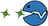	*T*_a_	No	Weaponry, escape behavior, robustness, autotomy
d) Digestion resistance (see Appendix S1)	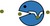	*T*_a_ + *T*_m_	No	Algae with thickened cell walls or snails surviving gut passage in predators

We also distinguish between two general cost types of the defense in respect to resource competition: either having a reduced performance at low resource concentrations or growing slower independent of the resource concentrations. Referring to the Monod equation (Monod, 1950), this corresponds either to a higher half‐saturation constant for resource uptake or a lower maximum growth rate. There is empirical evidence from phytoplankton and plant communities that both cost types are ecologically relevant (Agrawal, 1998; Lind et al., 2013; Meyer, Ellner, Hairston, Jones, & Yoshida, 2006; Yoshida, Hairston, & Ellner, 2004). However, a comparison of how both cost types affect the prey community is still missing.

Previous studies on predator‐mediated coexistence in diamond‐shaped food web models often implicitly assumed pre‐attack defenses (Abrams, 1999; Fryxell & Lundberg, 1994; Yamauchi & Yamamura, 2005) and considered either a higher half‐saturation constant (Becks et al., 2010; Jones & Ellner, 2007; Yoshida, Jones, Ellner, Fussmann, & Hairston, 2003) or a lower maximum growth rate as costs (Abrams, 1999; Kasada et al., 2014; Yoshida et al., 2007). In this study, we explicitly model pre‐attack and post‐attack defenses and both cost types. The modeled diamond‐shaped food web involves a basal resource, two competing prey types with a trade‐off between defense and competitiveness, and one predator species. One prey type is undefended, while the other type is defended either by a reduced probability of being attacked or a lower probability of being consumed when attacked. We consider defense as a continuous trait with values ranging from completely defended to nearly undefended. The costs for defense are either a higher half‐saturation constant or a lower maximum growth rate. By varying the values of both traits of the defended prey independently, we generate different magnitudes of the trade‐off quantifying the costs of being more or less defended. For each trait combination, we test for coexistence and check whether the populations cycle or are in steady state. This enables us to evaluate how the different traits promote maintenance of prey diversity and stabilize the dynamics. We analyze these effects under different enrichment levels (different resource concentrations) in order to vary the relative importance of bottom‐up and top‐down control.

## 2 METHODS

We consider a diamond‐shaped food web model with one predator (*P*), two prey types (*A*_*i*_), and one resource (*N*) limiting the growth of the prey. The two prey types face a trade‐off: *A*_1_ is defended but has costs in respect to resource competition while *A*_2_ is undefended and highly competitive. The following model description is divided into four parts. At first, we present the different defense mechanisms of *A*_1_ and derive the respective functional response of the predator. Second, we describe the different competition costs based on the resource‐dependent growth function of the prey types. Third, we apply the model to a fully parametrized chemostat system, and finally, we explain how to analyze the effect of the different defense mechanisms and costs on prey coexistence and the population dynamics for the considered system.

### 2.1 Defense mechanisms

The predator attacks the prey *A*_*i*_ with the probability *p*_*i*_ and then consumes the captured prey with the probability *q*_*i*_. While *A*_2_ is completely undefended (*p*_2_ = *q*_2_ = 1), *A*_1_ is able to defend against predation at different phases of the predation sequence (Bateman et al., 2014). We distinguish between two general defense mechanisms: pre‐attack defenses (*p*_1_ < 1) and post‐attack defenses (*q*_1_ < 1). A third special defense mechanism where the prey is attacked and consumed (*p*_1_ = *q*_1_ = 1) but survives passing the digestive system of the predator is investigated in Appendix S1.

According to Brodie, Formanowicz, and Brodie (1991), we assume that the defended prey is specialized only on one defense mechanisms, that is, if *p*_1_ < 1 then *q*_1_ = 1 and vice versa, as investing in one strategy reduces the fitness advantage of the other. The main difference between the defense mechanisms lies in how they affect the predator's functional response. We consider a Holling type II functional response of the predator implying that it spends a certain handling time for each attacked prey individual before it is able to attack the next prey item. Thus, the rate of consumption of the predator saturates with increasing prey density. The handling time comprises the time for attacking and capturing the prey (*T*_a_), and, if the prey is consumed, the time for manipulating and digesting it (*T*_m_). The two prey type version of the Holling disk equation is then given by

(1) $$F_{i}=\frac{ap_{i}q_{i}A_{i}}{1+ap_{1}\left(T_{a}+q_{1}T_{m}\right)A_{1}+ap_{2}\left(T_{a}+q_{2}T_{m}\right)A_{2}}$$

where *a* represents the encounter rate (Bateman et al., 2014; Holling, 1959; Rueffler, Van Dooren, & Metz, 2006). It should be mentioned here that the attack probability *p*_*i*_ scales the encounter rate *a* in the presented version of the type II functional response (Equation (1)). Therefore, the product of *a* and *p*_*i*_ can be interpreted as the effective attack rate on *A*_*i*_.

The key to understand the effects of the different defense mechanisms on the functional response of predator is the handling time spent per prey individual. A completely undefended prey individual demands the full handling time of the predator attacking and manipulating it, that is, *T*_a_ + *T*_m_ (Table 1a). A pre‐attack defended prey individual with *p*_1_ = 0 is not attacked and thus demands no handling time of the predator (Table 1b) allowing the predator to focus on the undefended prey. In contrast, for a post‐attack defended prey individual with *q*_1_ = 0, the predator invests the attack time *T*_a_ without making use out of it (Table 1c). The relative size of *T*_a_ compared to *T*_m_ determines how much the different prey types differ in their handling times. To approach this difference systematically, we replace *T*_a_ by *c*_a_ *T* and *T*_m_ by *c*_m_ *T* where *T* is the total handling time, *c*_a_ the fraction of *T* spent for attacking the prey, and *c*_m_ the fraction of *T* invested into manipulation which can be replaced by *c*_m_ = 1−*c*_a_. This leads us to

(2) $$F_{i}=\frac{ap_{i}q_{i}A_{i}}{1+ap_{1}\left(c_{a}T+q_{1}\left(1-c_{a}\right)T\right)A_{1}+ap_{2}\left(c_{a}T+q_{2}\left(1-c_{a}\right)T\right)A_{2}}.$$

If *c*_a_ has very low values, pre‐ and post‐attack defenses do not differ substantially in their effect on the predator, while high values of *c*_a_ imply high differences in handling times needed for a pre‐ and post‐attack defended prey (see Equation (2) and Table 1).

### 2.2 Defense costs with respect to competitiveness

An empirically well‐established resource‐dependent growth model is the Monod equation with the parameters maximum per capita growth rate β_*i*_ and half‐saturation constant *K*_*i*_, that is, the resource concentration where the growth rate reaches half of the maximum (Monod, 1950). The Monod equation is equivalent to a Holling type II functional response but is not restricted to predator—prey interactions and has been applied also to autotrophic organisms taking up nutrients (e.g., Becks et al., 2010; Raatz, Gaedke, & Wacker, 2017; Yoshida et al., 2003). The per capita growth rate of *A*_*i*_ in dependence of *N* is described by

(3) $$G_{i}=\beta_{i}\frac{N}{K_{i}+N}.$$

We distinguish here between two general types of defense costs of *A*_1_ with respect to competitiveness: a reduced growth rate at low but not at high resource concentrations (*K*_1_ > *K*_2_) or a lower growth rate independent of the resource concentration (β_1_ < β_2_). Both cost traits (*K*_1_ and β_1_) are relevant in nature, and their implications can be understood based on two extreme cases. First, for very high resource concentrations (*N* ≫* K*_1_), the per capita growth rate of *A*_1_ reaches its maximum and is independent of the half‐saturation constant (*G*_1_ = β_1_, see Equation (3)). Second, for very low resource concentrations (*N* ≪ *K*_1_), the per capita growth rate of *A*_1_ is given by $G_{1}=\left(\beta_{1}/K_{1}\right)N$ (see Equation (3)) and thus depends on both cost traits. According to that, β_*i*_/*K*_*i*_ can be interpreted as the slope of the growth function at very low *N* which is defined as the resource affinity. In the absence of mortality, the prey type with the higher resource affinity would be competitive superior (Button, 1978; Healey, 1980; Smith, Merico, Wirtz, & Pahlow, 2014). However, given a certain rate of natural mortality δ, the competitiveness depends on the equilibrium resource concentration $N_{i}^{*}$ at which the gross growth rate equals the mortality, that is, *G*_*i*_ = δ (Tilman, 1982). Following Equation (3), the equilibrium resource concentration of a prey type *A*_*i*_ in monoculture is given by

(4) $$N_{i}^{*}=\frac{K_{i}}{\frac{\beta_{i}}{\delta }-1}.$$

The undefended prey *A*_2_ has a lower equilibrium resource concentration than the defended prey *A*_1_ ($N_{2}^{*}<N_{1}^{*}$) and thus outcompetes *A*_1_ in the absence of predation. We use the ratio $N_{2}^{*}/N_{1}^{*}$ as a measure of relative competitiveness of *A*_1_ which allows us to compare the effects of the different defense costs (higher *K*_1_ or lower β_1_) on competition. For costs arising from *K*_1_ > *K*_2_ (β_1_ = β_2_), $N_{2}^{*}/N_{1}^{*}$ equals *K*_2_/*K*_1_. For costs originating from β_1_ < β_2_ (*K*_1_ = *K*_2_), it is given by $\left(\beta_{1}-\delta )/(\beta_{2}-\delta \right)$ (see Equation (4)).

### 2.3 Chemostat model

Here, we put the diamond‐shaped food web model with the previously derived functional responses and growth functions into an ecologically relevant context. We consider a chemostat system which is characterized by a continuous inflow of medium with resources and outflow of medium with resources and organisms (Smith & Waltman, 1995). The magnitude of the in‐ and outflow is described by the dilution rate δ which represents the mortality rate of the prey and the predator. The resource concentration in the supplied medium *N*_*I*_ determines the quantity of inflowing resources. An increase in *N*_*I*_ implies an enrichment of the system. The changes of the resource concentration and population densities over time are defined by the following differential equations

(5) $$\begin{array}{cc} \frac{dN}{dt} & =\delta \left(N_{I}-N\right)-\frac{1}{\chi }G_{1}A_{1}-\frac{1}{\chi }G_{2}A_{2}\\ \frac{dA_{i}}{dt} & =G_{i}A_{i}-F_{i}P-\delta A_{i}\\ \frac{dP}{dt} & =\chi_{P}F_{1}P+\chi_{P}F_{2}P-\delta P \end{array}$$

with *i *=* *1, 2. The parameter χ describes the prey's conversion efficiency of resources into prey individuals. The parameter χ_*P*_ describes the efficiency with which consumed prey individuals are converted into predator individuals. To reach a suitable parametrization, we refer our model to an empirically well‐studied rotifer‐algae system with *Brachionus calyciflorus* as a predator and different genotypes of *Chlamydomonas reinhardtii* as prey (Becks, Ellner, Jones, & Hairston, 2012; Becks et al., 2010). For details on the values and units of the parameters and the state variables, see Table 2. For the given system, *A*_1_ and *A*_2_ represent different genotypes of the same algal species. However, mechanistically, there is no difference between analyzing coexistence of different species or genotypes of an asexually reproducing species without horizontal gene transfer.

**Table 2:** Values and units of state variables and parameters used in the predator–prey chemostat model parametrized for a rotifer‐algae system (Becks et al., 2010, 2012)

Parameter/Variable	Value	Unit	References
*N*	–	μmol N/L	–
*A*_*i*_	–	ind./mL	–
*P*	–	ind./mL	–
*N*_*I*_	80, 160 or 240	μmol N/L	TVC
δ	0.8	per day	TVC
χ	2.7 × 10^6^	ind./μmol N	Lutz Becks, unpublished data
χ_*P*_	170 × 10^−6^	–	Becks et al. (2010)
*a*	0.073	mL/day	Calculated from Becks et al. (2010)
*T*	9.091 × 10^−5^	day	Calculated from Becks et al. (2010)
*c*_a_	0.5	–	NM
*p*_*i*_	*p*_1_ varied, *p*_2_ = 1.0	–	Lutz Becks, unpublished data
*q*_*i*_	*q*_1_ varied, *q*_2_ = 1.0	–	Lutz Becks, unpublished data
*K*_*i*_	*K*_1_ varied, *K*_2_ = 2.2	μmol N/L	Lutz Becks, unpublished data
β_*i*_	β_1_ varied, β_2_ = 1.6	per day	Noemi Woltermann, unpublished data

TVC, Typical values used in chemostat experiments with rotifers and algae as they enable sufficient rotifer densities but avoid light limitation (e.g., Becks et al., 2010; Yoshida et al., 2003); NM, No measurements available. For simplicity, we assume that the fraction of *T* spent for attacking and manipulating the prey is equal, that is, *c*_a_ = 0.5 (sensitivity analysis in Appendix S3).

### 2.4 Analysis of coexistence and population dynamics

The traits of the undefended prey *A*_2_ are fixed. By varying the defense level and the defense costs of *A*_1_ independently, we generate different slopes of the trade‐offs. In order to understand the individual effects of the different traits, we vary only the value of one defense trait and one cost trait at a time. The other trait values are equal to those of *A*_2_. This results in four different types of trade‐offs (TO) which we consider: p‐K‐TO, q‐K‐TO, p‐β‐TO, and q‐β‐TO. First, we analyze how the different defense mechanisms (*p*_1_ < *p*_2_ or *q*_1_ < *q*_2_) affect prey coexistence and the population dynamics, that is, whether cycles or steady state occur. Secondly, we compare the effect of different costs of defense (*K*_1_ > *K*_2_ or β_1_ < β_2_) on these properties.

In order to find the coexistence regions in the trait space, we applied an analytical approach of Jones and Ellner (2007) which calculates the condition for a coexistence equilibrium where all net growth rates equal zero. A following linear stability analysis of these equilibria informs about the population dynamics. A steady‐state occurs in case of a stable equilibrium. For an unstable equilibrium, coexistence with cycling population densities is possible. To check the basin of attraction of the respective attractor, that is, the range of possible initial conditions leading to it, we perform an invasion analysis. We check whether *A*_1_ can invade a resident community with *N*,* A*_2_ and *P* which reveals whether coexistence is also reached with very low population densities of *A*_1_. For further details on the analysis, see Appendix S2 in Supporting information. To investigate details of the population dynamics, we did numerical integrations for selected parameter combinations with the odeint solver of the SciPy package in Python (Jones, Oliphant, & Peterson, 2001). For all simulations shown in the main text, we use the same setting of initial population densities (*N* = *N*_*I*_, *A*_1_ = *A*_2_ = 10^5^ ind./mL, *P *=* *1 ind./mL) and a simulation time of 60 days.

## 3 RESULTS

### 3.1 General patterns of prey coexistence and population dynamics

**Figure 1:**
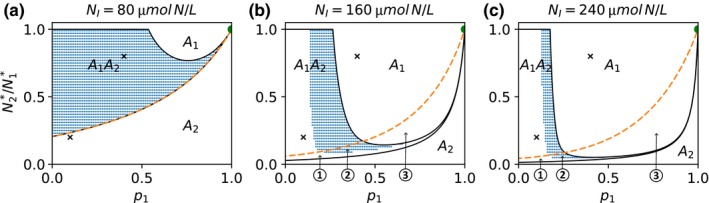
Prey coexistence and population dynamics for a trade‐off (TO) between pre‐attack defense, *p*, and half‐saturation constant *K* (p‐K‐TO) under three levels of resource supply, that is, *N*_*I*_ = 80, 160, 240 μmol N/L (a–c). The traits of the undefended prey *A*_2_ are kept constant (green dot). The attack probability *p*_1_ and the half‐saturation constant *K*_1_ of the defended prey *A*_1_ which determines its relative competitiveness $N_{2}^{*}/N_{1}^{*}$ (cf. Methods, Equation (4)) are varied within the shown trait space. The capital letters display which prey types survive in the different parts of the trait space. The black lines enclose the part of the trait space where a coexistence equilibrium exists while blue dots mark where it is locally stable (steady state). The dashed orange line represents the invasion boundary above which *A*_1_ can invade a resident community with *A*_2_. The numbers indicate special cases which occur at intermediate and high enrichment levels (b, c): ➀ Only *A*_2_ survives as the coexistence equilibrium is unstable and *A*_1_ cannot invade, ➁ multistability between coexistence and survival of only *A*_2_, ➂ multistability between survival of *A*_1_ and survival of *A*_2_. The crosses mark trait combinations used in Figure 5

To explain the patterns of coexistence and population dynamics in general, we focus initially on the trade‐off (TO) between attack probability *p*, that is, pre‐attack defense, and half‐saturation constant *K* (p‐K‐TO). We consider three different enrichment levels, that is, *N*_*I*_ = 80, 160, and 240 μmol N/L, to reveal the sensitivity of these patterns to the productivity of the system (Figure 1a–c).

For a low resource supply (*N*_*I*_ = 80 μmol N/L), the defended prey *A*_1_ dies out and only the undefended prey *A*_2_ survives if the relative competitiveness $N_{2}^{*}/N_{1}^{*}$ (cf. Methods, Equation (4)) of *A*_1_ is very low, that is, *A*_1_ has a high half‐saturation constant *K*_1_ (Figure 1a). For higher values of $N_{2}^{*}/N_{1}^{*}$, *A*_1_ and *A*_2_ may coexist. The range of possible $N_{2}^{*}/N_{1}^{*}$ values enabling coexistence increases with a decreasing attack probability *p*_1_ (Figure 1a). At low values of *p*_1_, both prey types coexist for relatively low $N_{2}^{*}/N_{1}^{*}$ values and even for high $N_{2}^{*}/N_{1}^{*}$ values close to 1 (very low defense costs). The latter case implies a very high fitness of *A*_1_ as it has favorable values for both traits. However, such a highly defended *A*_1_ cannot outcompete *A*_2_ as long as its competitiveness is lower than that of *A*_2_ ($N_{2}^{*}/N_{1}^{*}<1$) because *A*_1_ needs *A*_2_ to maintain the predator *P* and thus its advantage of being defended. In the absence of *P*,* A*_1_ would be inferior compared to *A*_2_ as long as its competitiveness is slightly lower than that of *A*_2_. At high values of *p*_1_, *A*_1_ can maintain *P* by itself for $N_{2}^{*}/N_{1}^{*}$ close to 1 and thus outcompetes *A*_2_ (Figure 1a).

For low levels of resource supply (*N*_*I*_ = 80 μmol N/L), both prey types coexist in a steady state; that is, the coexistence equilibrium is always stable (Figure 1a). Furthermore, the outcome of coexistence is independent of the initial conditions as the invasion boundary of *A*_1_ is identical to the boundary of the region where coexistence equilibria exist, indicating that they are globally stable. This pattern changes if we enrich the system, that is, increase *N*_*I*_ to 160 or 240 μmol N/L. A higher resource supply enhances the occurrence of coexistence in cycles, that is, unstable coexistence equilibria (Figure 1b,c). The higher concentration of resources reduces the bottom‐up control which promotes the fitness of *A*_1_ as its disadvantage regarding competition for resources gets less important. Therefore, the part of the trait space where *A*_1_ goes extinct decreases while the part where *A*_1_ outcompetes *A*_2_ strongly increases which alters also the trait space of coexistence (Figure 1b,c). Moreover, the invasion boundary of *A*_1_ is not identical to the boundary of coexistence equilibria any more, implying that even if a coexistence equilibrium exists, the two prey types may not coexist. In parts of the trait space below the invasion boundary of *A*_1_ where the coexistence equilibrium is unstable, there is no attractor enabling coexistence and only *A*_2_ survives (➀ in Figure 1b,c). In the region where a locally stable coexistence equilibrium exists but *A*_1_ cannot invade (➁ in Figure 1b,c), either both prey types coexist or *A*_1_ goes extinct depending on the initial conditions. Multistability occurs also at trait ranges above the region of coexistence equilibria where *A*_1_ cannot invade *A*_2_ (➂ in Figure 1b,c). Here, priority effects matter: either *A*_1_ outcompetes *A*_2_ when present at initially high densities or goes extinct when *A*_2_ is initially dominant (for details on multistability see Appendix S4).

The predator *P* survives in all trait areas stated above for all enrichment levels. It would only die out when a highly defended *A*_1_ has a higher competitiveness than *A*_2_ ($N_{2}^{*}/N_{1}^{*}>1$) leading to the extinction of *A*_2_, the only suitable food source of *P* in this case.

### 3.2 Comparison of different defense mechanisms

To demonstrate the effects of the different defense mechanisms on coexistence and population dynamics, we chose an intermediate resource supply (*N*_*I*_ = 160 μmol N/L) where coexistence with population cycles occurs and the defended prey *A*_1_ is able to outcompete the undefended prey *A*_2_ for a significant part of the trait space (Figure 1b). The results for *N*_*I*_ = 80 and 240 μmol N/L are given in Appendix S3 (Figures C1 and C2).

**Figure 2:**
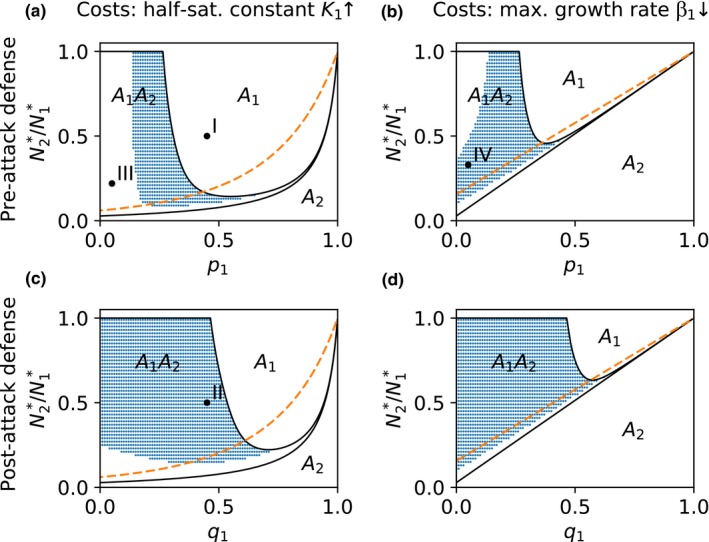
Comparison of different types of trade‐offs between defense and resource competition regarding their effect on coexistence and population dynamics of a defended prey *A*_1_ and an undefended prey *A*_2_ at an intermediate resource supply (*N*_*I*_ = 160 μmol N/L). The trait values of *A*_1_ are varied within the shown trait space. The defense occurs either prior to an attack by a predator via a lower attack probability *p*_1_ (a, b) or after being attacked by a lower consumption probability *q*_1_ (c, d). The costs of the defense are either a higher half‐saturation constant *K*_1_ (a, c) or a reduced maximum growth rate β_1_ (b, d). Both cost traits affect the relative competitiveness $N_{2}^{*}/N_{1}^{*}$ of *A*_1_ compared to *A*_2_ (cf. Methods, Equation (4)). The capital letters display main regions in the trait space with different competition outcomes: Only the undefended prey survives (*A*_2_), only the defended prey survives (*A*_1_), or both prey types coexist (*A*_1_ *A*_2_). Coexistence equilibria exist within the region surrounded by black lines, while blue dots indicate where they are locally stable. The dashed orange line marks the invasion boundary of *A*_1_. For further details on other regions with multistability, see Figure 1. The black dots with Roman numerals mark trait combinations for which population dynamics and growth functions are shown in Figure 3 (I, II) and Figure 4 (III, IV)

**Figure 3:**
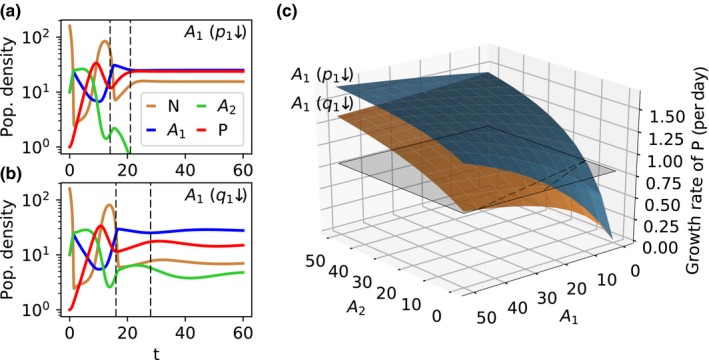
(a) Population dynamics for a pre‐attack defense–half‐saturation constant trade‐off (trait combination I in Figure 2a, *p*_1_ = 0.45 and *K*_1_ = 4.4 μmol N/L) and (b) a post‐attack defense–half‐saturation constant trade‐off (trait combination II in Figure 2c, *q*_1_ = 0.45 and *K*_1_ = 4.4 μmol N/L). The population densities of the resource *N* (μmol N/L), the two prey types *A*_*i*_ (10^4^ ind./mL), and the predator *P* (ind./mL) are plotted over time *t* (day). The vertical dashed lines enclose one growing phase of *P*. (c) Per capita growth rate of *P* without mortality in dependence of the densities of the defended prey *A*_1_ and the undefended prey *A*_2_. The blue surface shows the growth function when *A*_1_ is pre‐attack defended (like in a), while the orange surface represents the growth function in case of a post‐attack defended *A*_1_ (like in b). The post‐attack defense reduces the growth rate of *P* by keeping it unprofitably handling with defended prey individuals while there is no handling of pre‐attack defended prey. The gray surface shows the dilution rate which represents the mortality of the predator. The black dashed lines illustrate where the growth rate of *P* equals its mortality, that is, where its net growth rate is zero

Comparing the different types of defense–competition trade‐offs, we find qualitatively the same regions of coexistence in the trait space but with greatly differing importance (Figure 2a–d). A post‐attack defense (*p*_1_ = 1, *q*_1_ < 1) promotes coexistence more strongly than a pre‐attack defense (*p*_1_ < 1, *q*_1_ = 1) and stabilizes the dynamics. This observation is independent of the type of costs for the defense (Figure 2a–d). We elucidate the effects of the different defense mechanisms based on the comparison of the attack probability–half‐saturation constant trade‐off (p‐K‐TO, Figure 2a) and the consumption probability–half‐saturation constant trade‐off (q‐K‐TO, Figure 2c). The part of the trait space where the defended prey *A*_1_ outcompetes the undefended prey *A*_2_ is smaller for the q‐K‐TO while the coexistence region increases (Figure 2a,c). The region where *A*_1_ goes extinct remains constant. The changes in the coexistence patterns can be explained with the different growth functions of the predator *P* resulting from the different defense mechanisms. We consider two comparable levels of the different defenses (*p*_1_ = 0.45 or *q*_1_ = 0.45) having the same costs ($N_{2}^{*}/N_{1}^{*}=0.5$). The population dynamics clearly reveal that the growth rate of *P* is higher for the p‐K‐TO compared to the q‐K‐TO although the amount of total available prey (Σ*p*_*i*_ *q*_*i*_ *A*_*i*_) is slightly lower during the predator growing phase (Figure 3a,b). The resulting higher biomass of *P*, that is, the higher top‐down control, in case of the p‐K‐TO drives the undefended prey *A*_2_ to extinction (Figure 3a).

More generally, the growth rates of *P* are equal for the p‐K‐TO and the q‐K‐TO in the absence of *A*_1_, but they diverge for the different types of defenses with an increasing density of the defended prey *A*_1_ (Figure 3c). With higher shares of *A*_1_, the predator is increasingly handling “noncrackable” prey for the q‐K‐TO which dampens its growth in comparison with the p‐K‐TO. At high densities of *A*_2_, an increasing density of *A*_1_ leads even to a reduction of growth in case of the q‐K‐TO, while there is a slight increase in growth for the p‐K‐TO (Figure 3c). When *A*_2_ is absent, *P* dies out in case of the q‐K‐TO because the gross growth rate of *P* based on a post‐attack defended *A*_1_ lies below the mortality rate even for high densities of *A*_1_ resulting in a negative net growth of *P* (Figure 3c). Therefore, *A*_1_ cannot outcompete *A*_2_ as it needs *A*_2_ to maintain the predator and thus its advantage in respect to defense. In addition, *A*_1_ indirectly facilitates *A*_2_ as it reduces the grazing loss of *A*_2_ by keeping the predator handling “noncrackable” food items (Figure 3c). The described effects of the post‐attack defense on predator growth result in the coexistence of both prey types for an extended trait space. These effects are absent in case of the pre‐attack defense leading to the extinction of *A*_2_. The lower growth rates of *P* in case of the q‐K‐TO explain also the more frequent occurrence of steady states (Figure 2c). Cycles require sufficient deflections of population densities, that is, high minima of prey densities, which are prevented due to the lower growth rates of *P*.

### 3.3 Comparison of different defense costs

**Figure 4:**
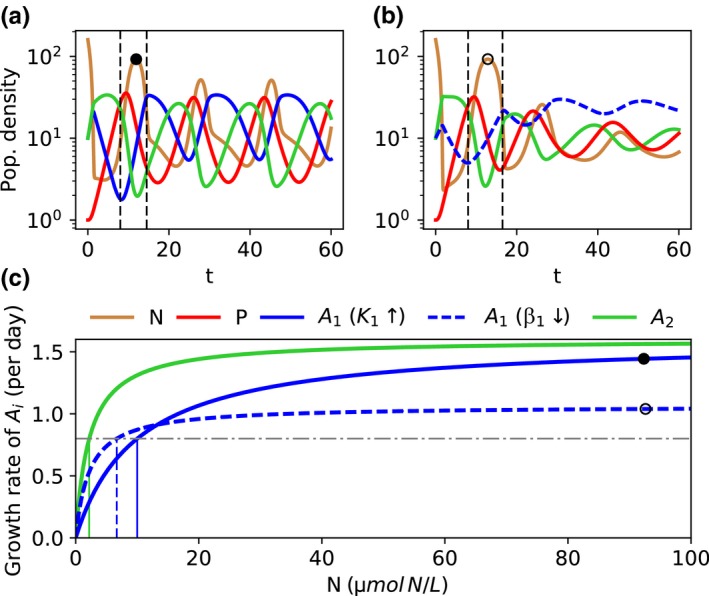
(a) Population dynamics for a trade‐off between pre‐attack defense and half‐saturation constant (trait combination III in Figure 2a) or (b) maximum growth rate (trait combination IV in Figure 2b). The population densities of the resource *N* (μmol N/L), both prey types *A*_*i*_ (10^4^ ind./mL) and the predator *P* (ind./mL) are plotted over time *t* (*d*). The pre‐attack defended prey *A*_1_ (*p*_1_ = 0.05) has either a higher half‐saturation constant (*K*_1_ = 10 μmol N/L) (a) or a reduced maximum growth rate (β_1_ = 1.064 per day) (b) compared to the undefended prey *A*_2_. The black dashed lines enclose one growing phase of *A*_1_ at high resource concentrations (peak marked with dot) after strong grazing of *A*_2_ by *P*. (c) Per capita growth rates of the prey types without mortality in dependence of the resource concentration are represented by the thick lines. The thin vertical green and blue lines show the resource concentration at equilibrium in monoculture $N_{i}^{*}$ of each prey type where lower values of $N_{i}^{*}$ imply higher competitiveness. The horizontal black dashed‐dotted line represents the dilution rate, that is, the mortality of the prey without the predator. The dots illustrate the realized growth rate of the respective defended prey at the resource peaks shown in (a, b)

We now compare the effects of different cost types based on the trade‐off between attack probability and half‐saturation constant (p‐K‐TO) or maximum growth rate (p‐β‐TO). A higher half‐saturation constant *K*_1_ rather than a lower maximum growth rate β_1_ as defense costs allows the defended prey *A*_1_ to survive even at lower values of relative competitiveness $N_{2}^{*}/N_{1}^{*}$ and promotes cycles more strongly at high and intermediate defense levels, that is, low and intermediate *p*_1_ (Figure 2a,b). The advantage of *A*_1_ facing a p‐K‐TO and the altered stability of coexistence equilibria can be explained based on the population dynamics and growth functions of the prey types shown in Figure 4 for the trait combinations (III, IV) marked in Figure 2a,b.

For the p‐K‐TO, the system cycles (Figure 4a) while damped oscillation occur at the shown p‐β‐TO (Figure 4b). The trait combinations III, IV are chosen as they lead to a similar level of resource concentrations and predator densities in the first growing phase of *A*_1_ (marked phase in Figure 4a,b). Despite the similar environmental conditions in this phase, *A*_1_ reaches a much higher growth rate for the p‐K‐TO than for the p‐β‐TO (Figure 4a–c). In general, under (at least transient) cyclic conditions, *A*_1_ increases in density when the predator strongly consumes *A*_2_ leading to an increased resource availability (Figure 4a,b). Accordingly, *A*_1_ grows at a resource peak. At high resource concentrations, the growth function of *A*_1_ is getting close to that of *A*_2_ for the p‐K‐TO as they have the same maximum growth rate, but it remains consistently lower in case of the p‐β‐TO (Figure 4c). The higher growth rate of *A*_1_ in case of the p‐K‐TO leads to more unstable equilibria and increases the occurrence of cycles compared to the p‐β‐TO. This explains also why *A*_1_ survives also for a lower competitiveness $N_{2}^{*}/N_{1}^{*}$ over a large range of *p*_1_ values in case of the p‐K‐TO. Costs regarding *K*_1_ can be seen as a more temporal disadvantage which only becomes relevant during pronounced resource depletion, that is, bottom‐up control. During top‐down control (high predator densities), this disadvantage is less important as the resource conditions are good. The lower competitiveness of *A*_1_ under resource depletion is counteracted by the relatively high growth rate at resource peaks enabling it to survive.

We find basically the same effects of the different cost types when considering the other defense mechanism with the reduced consumption probability (q‐K‐TO and q‐β‐TO). However, the reduced occurrence of cycles in case of the β‐costs is less evident as the post‐attack defense mechanism is already stabilizing (Figure 2c,d).

### 3.4 The effect of the enrichment level

**Figure 5:**
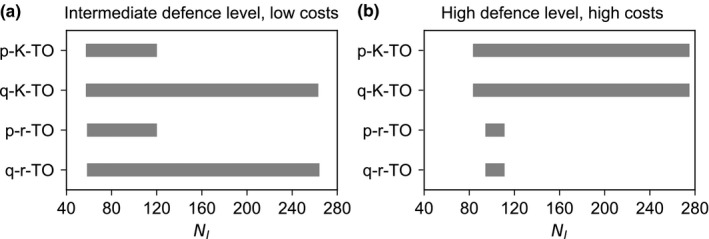
Coexistence of two prey types depending on the level of resource supply *N*_*I*_ (in μmol N/L), that is, the enrichment level, for different trade‐offs (TO) between pre‐attack (*p*) or post‐attack defense (*q*) and the half‐saturation constant (*K*) or maximum growth rate (β). The bars indicate the range where both prey types stably coexist, implying that a coexistence equilibrium exists and the defended prey can invade the undefended prey. The defended prey has either (a) an intermediate defense level and low costs (*p*_1_ or *q*_1_ = 0.4, $N_{2}^{*}/N_{1}^{*}=0.8$) or (b) a high defense level and high costs (*p*_1_ or *q*_1_ = 0.1, $N_{2}^{*}/N_{1}^{*}=0.2$). These trait combinations are indicated in Figure 1

So far, we analyzed how the different defense mechanisms and costs affect the proportion of the trait space leading to coexistence at a certain resource supply (Figure 2). Now, we fix the trait values of both prey types but vary the enrichment level (*N*_*I*_) to evaluate the maintenance of coexistence under altered environmental conditions. Pre‐attack and post‐attack defenses differ in their implications for coexistence within a broad range of intermediate defense levels (Figure 2). The type of costs is most relevant at high cost levels and intermediate to high defense levels (Figure 2). Accordingly, we chose such trait combinations of the defended prey, that is, an intermediate defense level with low costs and a high defense level with high costs (marked in Figure 1), to examine how the differences in coexistence patterns depend on the enrichment level (Figure 5a,b).

At an intermediate level of defense with low costs, coexistence is promoted by the post‐attack defense in comparison with the pre‐attack defense, independent of the type of costs (Figure 5a). The post‐attack defense allows the prey types to stably coexist over a wide range of enrichment levels from low to very high resource supplies while in case of the pre‐attack defense coexistence is only possible for a low resource supply (Figure 5a). Contrastingly, at a high level of defense and costs, the coexistence patterns diverge between the different cost types but are rather independent from the defense mechanism. Costs with respect to the half‐saturation constant enable coexistence for lower and especially higher enrichment levels compared to costs regarding the maximum growth rate (Figure 5b).

### 3.5 Sensitivity analysis

In Appendix S3, we show how the coexistence and population dynamics of the prey types depend on the amount of the attack time relative to the manipulation time which is defined by *c*_a_ (fraction of total handling time spent for attacking). With higher values of *c*_a_, the post‐attack defense increasingly promotes coexistence and stabilizes the dynamics, while the results for the pre‐attack defense are independent of *c*_a_ (Appendix S3: Figures C3 and C4). Furthermore, we check the sensitivity of our results with respect to the encounter rate *a*, the total handling time *T*, the conversion efficiency of the predator χ_*P*_, the conversion efficiency of the prey χ, the resource concentration in the supplied medium *N*_*I*_, the dilution rate δ, the maximum growth rate of the undefended prey β_2_, and its half‐saturation constant *K*_2_. The general pattern that post‐attack defenses promote coexistence and stabilize the dynamics compared to pre‐attack defenses is independent of these parameter values. The same holds for the observed pattern that costs regarding the half‐saturation constant promote coexistence and the occurrence of cycles more strongly than costs with respect to the maximum growth rate (Appendix S3: Figures C5–C7). Obviously, the exact trait values for the occurrence of coexistence equilibria and their local stability depend on the values of all parameters.

## 4 DISCUSSION

We compared the effects of pre‐attack and post‐attack defenses with different costs in respect to resource competition on coexistence and population dynamics in a diamond‐shaped food web. The post‐attack defense promoted coexistence and stabilized the dynamics more than a pre‐attack defense. Post‐attack defended individuals damped the growth of the predator by keeping it handling them which indirectly facilitates the undefended prey. This mechanism enabled coexistence at trait combinations where the defended prey would outcompete the undefended one in case of a pre‐attack defense. Costs regarding resource competition were either a higher half‐saturation constant or a lower maximum growth rate. The former cost type promoted coexistence and cycling population densities more than a lower maximum growth rate by allowing the defended prey to realize high growth rates at temporally high resource concentrations which prevents its extinction even if it has a very low competitiveness.

The main difference between both defense mechanisms is that a post‐attack defended prey affects the functional response of the predator consuming an undefended prey while a pre‐attack defended prey does not. In a previous study of Grover (1995), inedible plants were generally described as interfering when they negatively affect the growth of the herbivore consuming an edible plant. This interference weakens the interaction between the predator and its prey. In food web theory, weak interactions are known to stabilize the population dynamics (McCann, 2011; McCann, Hastings, & Huxel, 1998). In fact, several authors (Grover, 1995; Kretzschmar, Nisbet, & Mccauley, 1993; Vos, Berrocal, Karamaouna, Hemerik, & Vet, 2001) showed that interfering inedible species stabilized dynamics in comparison with noninterfering inedible ones which resembles the effect observed in our study, while they did not reveal the potential of interfering defended prey to enhance coexistence. This was not possible in their systems because the defended prey was completely inedible. Thus, it could not maintain the predator by itself and always had to coexist with the undefended prey. Contrastingly, we considered defense as a continuous trait. At low and intermediate defense levels, the defended prey is able to outcompete the undefended prey. The trait range where this competitive exclusion of the undefended prey occurred was strongly reduced by the post‐attack defense, while the trait range allowing coexistence increased in comparison with a noninterfering pre‐attack defense due to indirect facilitation. The relevance of our results is supported by empirical studies revealing that low to intermediate defense levels frequently occur in nature (White, Kaul, Knoll, Wilson, & Sarnelle, 2011) and that defended prey types may outcompete undefended prey types even if they have costs for their defense (Kasada et al., 2014). The presented mechanism of indirect facilitation among prey species may provide an explanation why apparent competition, that is, an increasing density of one prey species indirectly reduces the density of the other prey species via the predator (Holt, 1977), is not always observed in nature (Chaneton & Bonsall, 2000).

If attacking defended prey types hampers predator's growth, the question arises whether the predator adapts and entirely disregards the defended prey implying that post‐attack defenses become pre‐attack defenses. Pre‐attack defenses are also favorable from the point of view of the defended prey which is then able to better outcompete a competing undefended prey. Accordingly, predator and defended prey may evolve toward avoiding interactions with each other which would mean that being not attacked is the prevailing defense strategy. This reasoning is supported by the observation that many defended prey species show warning signals to deter the predator from attacking them (Blount et al., 2012; Stevens & Ruxton, 2012) and that predators often show behavioral changes to avoid defended prey species (White et al., 2011; Xu, Nielsen, & Kiørboe, 2018). However, not attacking defended prey species implies a higher grazing pressure on undefended prey species which may reduce their population densities leading to the dominance of defended prey species. The resulting lack in food may cause predator attacks on the defended prey (Barnett, Bateson, & Rowe, 2007; Fryxell & Lundberg, 1994). However, if the defense of the prey is very effective, such prey switching behavior of the predator is unlikely. Several studies highlighted the importance of the interplay between the predator's diet choice and the level of defense for prey coexistence and population dynamics (Abrams & Matsuda, 1993; Fryxell & Lundberg, 1994; Yamauchi & Yamamura, 2005). Another argument for the occurrence of post‐attack defenses in nature is the inability of predators to discriminate between undefended and defended prey. This may hold especially for predators with nonselective feeding strategies, like, for example, filter feeding *Daphnia* sp. consuming filamentous algae (Peter & Lampert, 1989). Furthermore, it strongly depends on the costs of the prey which defense mechanisms evolve (Bateman et al., 2014). Post‐attack defenses may evolve if pre‐attack defenses are very costly, for example, if avoiding habitats with predators substantially lowers the possibility of resource acquisition (Verdolin, 2006).

The used categorization into pre‐ and post‐attack defenses is based on mechanistic considerations regarding their effect on the invested handling time of the predator. Even if these categories are relevant for a broad range of defense mechanisms, they may not apply to every specific defense strategy. For example, several algal species are digestion‐resistant; that is, they are attacked and ingested by the zooplankton but survive the gut passage (Demott & McKinney, 2015; Meyer et al., 2006; Porter, 1973). The same holds also for some species of aquatic snails eaten by mallards (van Leeuwen, van der Velde, van Lith, & Klaassen, 2012; Wada, Kawakami, & Chiba, 2012). Such digestion resistance may promote coexistence and stabilize the dynamics even more than post‐attack defended prey (see Appendix S1). Furthermore, toxicity of a prey may differ in the consequences on prey coexistence from the defense mechanisms considered here, especially when it interferes not only with the predator but also with the competitor (Hiltunen, Barreiro, & Hairston, 2012). However, for a large variety of defense strategies, for example, aposematism, weaponry or mimicry, the used classification regarding the phase at which the defense interrupts the predation sequence is adequate. We distinguished between early (pre‐attack) and late (post‐attack) defenses (Bateman et al., 2014). Of course, in nature there are gradients between pre‐ and post‐attack defenses, that is, the handling of the predator aborts at different points in time which can be mimicked by varying the parameter *c*_a_ (see Appendix S3). In the presented model, *c*_a_ was defined as the fraction of handling time invested in attacking prey. However, in a more general sense, *c*_a_ can be interpreted as the timing of a defended prey to abort the predation sequence relative to the total handling time, that is, a large *c*_a_ corresponds to a late defense and vice versa. Therefore, the given equation of the predator's functional response for two prey types (Equation (2)) can be applied to multiple defense mechanisms acting at different phases of the predation sequence.

Hammill, Kratina, Vos, Petchey, and Anholt (2015) provided first empirical evidence that inedible prey species promote persistence of edible ones based on an experiment with a flatworm feeding on ciliates. However, despite the wide‐spread occurrence of post‐attack defenses, we found no study specifically analyzing their effect on coexistence and comparing it to pre‐attack defenses. Thus, our research may serve as a starting point for future empirical studies on the maintenance of functional diversity within prey communities due to indirect facilitation of undefended prey species by post‐attack defended prey species.

Quantifying defense costs is often difficult as it requires knowledge about the functional property of the prey which is influenced by an altered allocation of resources to implement the defense. Furthermore, the costs may be system specific and may vary depending on the environment (Siemens, Garner, Mitchell‐Olds, & Callaway, 2002; Strauss, Rudgers, Lau, & Irwin, 2002); for example, they may occur only when a competitor is present (van Velzen & Etienne, 2015). We focussed on two major cost traits of defended prey types which describe their resource‐dependent growth kinetics: a higher half‐saturation constant which implies a reduced competitiveness at low resource concentration, and a lower maximum growth rate which reduces growth independent of the resource concentrations. Studies on plankton organisms revealed that trade‐offs between maximum growth rates and defenses frequently occur (Agrawal, 1998; Meyer et al., 2006) but half‐saturation constant‐defense trade‐offs were found as well (Becks et al., 2010; Yoshida et al., 2004). Furthermore, there is indication from phytoplankton organisms that the maximum growth rate and the half‐saturation constant are often positively correlated (Edwards, Klausmeier, & Litchman, 2013; Litchman, Edwards, & Klausmeier, 2015). Thus, Aksnes and Egge (1991) and Smith et al. (2014) suggested an alternative mechanistic formulation of nutrient‐uptake kinetics for phytoplankton organisms which accounted implicitly for this correlation. They used the affinity, that is, the slope of the uptake function at resource concentrations close to zero, instead of the half‐saturation constant as a parameter describing the performance at low resource concentrations. However, the outcome would be similar: a lower affinity rather than a lower maximum growth rate as defense costs promotes coexistence and destabilizes the dynamics because it allows for temporally high growth rates of the defended prey at resource peaks.

In the context of plant communities, there is an ongoing debate on the costs of defenses against herbivory. Several studies indicated that there is often no interspecific trade‐off between defense and competitiveness as a higher resource supply adversely affected defended plants (Lind et al., 2013; Viola et al., 2010). In fact, Lind et al. (2013) demonstrated that defended plants are commonly the better competitor when resources are depleted but perform less well at high resource concentrations. Such multidimensional trade‐offs may be included in future studies considering specific prey communities and their cost type/s of defense.

The coexistence of defended and undefended prey types critically depends on the relative importance of bottom‐up and top‐down control, that is, the enrichment level of the system (Bohannan & Lenski, 2000; Leibold, 1996; Proulx & Mazumder, 1998). Higher enrichment levels promote the defended prey as its disadvantage regarding resource competition gets less important relative to its advantage of being defended against predation. Thus, our insights on how the competitive exclusion of an undefended prey by an intermediately defended prey is prevented in case of post‐attack defense may prevail more in systems with an intermediate or high resource supply (Figure 5a). A high system productivity reduces also the potential extinction risk of the defended prey if the defense costs arise from a high half‐saturation costs where resource peaks permit temporally high growth rates of the defended prey (Figure 5b). Hence, we conclude that the current trend of anthropogenic eutrophication of ecosystems enhances the necessity to discriminate between different defense mechanisms and their associated costs.

## CONFLICT OF INTEREST

None declared.

## AUTHORS’ CONTRIBUTION

EE had the principal idea; EE and UG designed the study; EE did the mathematical and numerical analyses and wrote the manuscript on which UG commented.

## References

[ece34145-bib-0001] Abrams, P. A. (1999). Is predator‐mediated coexistence possible in unstable systems? Ecology, 80(2), 608–621.

[ece34145-bib-0002] Abrams, P. , & Matsuda, H. (1993). Effects of adaptive predatory and anti‐predator behaviour in a two‐prey‐one‐predator system. Evolutionary Ecology, 7(3), 312–326. 10.1007/BF01237749

[ece34145-bib-0003] Agrawal, A. A. (1998). Algal defense, grazers, and their interactions in aquatic trophic cascades. Acta Oecologica, 19(4), 331–337. 10.1016/S1146-609X(98)80037-4

[ece34145-bib-0004] Aksnes, D. , & Egge, J. (1991). A theoretical model for nutrient uptake in phytoplankton. Marine Ecology Progress Series, 70(1), 65–72. 10.3354/meps070065

[ece34145-bib-0005] Barnett, C. , Bateson, M. , & Rowe, C. (2007). State‐dependent decision making: Educated predators strategically trade off the costs and benefits of consuming aposematic prey. Behavioral Ecology, 18(4), 645–651. 10.1093/beheco/arm027

[ece34145-bib-0006] Bateman, A. W. , Vos, M. , & Anholt, B. R. (2014). When to defend: Antipredator defenses and the predation sequence. The American Naturalist, 183(6), 847–855. 10.1086/675903 24823827

[ece34145-bib-0007] Becks, L. , Ellner, S. P. , Jones, L. E. , & Hairston, N. G. Jr (2010). Reduction of adaptive genetic diversity radically alters eco‐evolutionary community dynamics. Ecology Letters, 13(8), 989–997. 20528898 10.1111/j.1461-0248.2010.01490.x

[ece34145-bib-0008] Becks, L. , Ellner, S. P. , Jones, L. E. , & Hairston, N. G. Jr (2012). The functional genomics of an eco‐evolutionary feedback loop: Linking gene expression, trait evolution, and community dynamics. Ecology Letters, 15(5), 492–501. 10.1111/j.1461-0248.2012.01763.x 22417636

[ece34145-bib-0009] Blount, J. D. , Rowland, H. M. , Drijfhout, F. P. , Endler, J. A. , Inger, R. , Sloggett, J. J. , … Speed, M. P. (2012). How the ladybird got its spots: Effects of resource limitation on the honesty of aposematic signals. Functional Ecology, 26(2), 334–342. 10.1111/j.1365-2435.2012.01961.x

[ece34145-bib-0010] Bohannan, B. J. M. , & Lenski, R. E. (2000). The relative importance of competition and predation varies with productivety in a model community. American Naturalist, 156(4), 329–340. 10.1086/303393 29592139

[ece34145-bib-0011] Brodie, E. , Formanowicz, D. , & Brodie, E. (1991). Predator avoidance and antipredator mechanisms: Distinct pathways to survival. Ethology Ecology & Evolution, 3(1), 73–77. 10.1080/08927014.1991.9525390

[ece34145-bib-0012] Button, D. (1978). On the theory of control of microbial growth kinetics by limiting nutrient concentrations. Deep‐Sea Research, 25(12), 1163–1177. 10.1016/0146-6291(78)90011-5

[ece34145-bib-0013] Chaneton, E. J. , & Bonsall, M. B. (2000). Enemy‐mediated apparent competition: Empirical patterns and the evidence. Oikos, 88(2), 380–394. 10.1034/j.1600-0706.2000.880217.x

[ece34145-bib-0014] Chase, J. M. , Abrams, P. A. , Grover, J. P. , Diehl, S. , Chesson, P. , Holt, R. D. , … Case, T. J. (2002). The interaction between predation and competition: A review and synthesis. Ecology Letters, 5, 302–315. 10.1046/j.1461-0248.2002.00315.x

[ece34145-bib-0015] Ciros‐Pérez, J. , Carmona, M. J. , Lapesa, S. , & Serra, M. (2004). Predation as a factor mediating resource competition among rotifer sibling species. Limnology and Oceanography, 49(1), 40–50. 10.4319/lo.2004.49.1.0040

[ece34145-bib-0016] Coley, P. D. , Bryant, J. P. , & Chapin, F. S. (1985). Resource availability and plant antiherbivore defense. Science, 230(4728), 895–899. 10.1126/science.230.4728.895 17739203

[ece34145-bib-0017] Demott, W. R. , & McKinney, E. N. (2015). Use it or lose it? Loss of grazing defenses during laboratory culture of the digestion‐resistant green alga Oocystis. Journal of Plankton Research, 37(2), 399–408. 10.1093/plankt/fbv013

[ece34145-bib-0018] Edwards, K. F. , Klausmeier, C. A. , & Litchman, E. (2013). A three‐way trade‐off maintains functional diversity under variable resource supply. The American Naturalist, 182(6), 786–800. 10.1086/673532 24231539

[ece34145-bib-0019] Ehrlich, E. , Becks, L. , & Gaedke, U. (2017). Trait‐fitness relationships determine how trade‐off shapes affect species coexistence. Ecology, 98(12), 3188–3198. 10.1002/ecy.2047 29034456

[ece34145-bib-0020] Endler, J. A. (1991). Interactions between predators and prey In Krebs J. R. & Davies N. B. (Eds.), Behavioral ecology: An evolutionary approach (3rd ed., pp. 169–196). Oxford, UK: Blackwell.

[ece34145-bib-0021] Fauth, J. E. , & Resetarits, W. J. (1991). Interactions between the salamander siren intermedia and the keystone predator notophthalmus viridescens. Ecology, 72(3), 827–838. 10.2307/1940585

[ece34145-bib-0022] Fryxell, J. M. , & Lundberg, P. (1994). Diet choice and predator‐prey dynamics. Evolutionary Ecology, 8(4), 407–421. 10.1007/BF01238191

[ece34145-bib-0023] Genkai‐Kato, M. , & Yamamura, N. (1999). Unpalatable prey resolves the paradox of enrichment. Proceedings of the Royal Society B: Biological Sciences, 266(1425), 1215–1219. 10.1098/rspb.1999.0765

[ece34145-bib-0024] Grover, J. P. (1995). Competition, herbivory, and enrichment: Nutrient‐based models for edible and inedible plants. The American Naturalist, 145(5), 746–774. 10.1086/285766

[ece34145-bib-0025] Hammill, E. , Kratina, P. , Vos, M. , Petchey, O. L. , & Anholt, B. R. (2015). Food web persistence is enhanced by non‐trophic interactions. Oecologia, 178(2), 549–556. 10.1007/s00442-015-3244-3 25656586

[ece34145-bib-0026] Healey, F. P. (1980). Slope of the Monod equation as an indicator of advantage in nutrient competition. Microbial Ecology, 5(4), 281–286. 10.1007/BF02020335 24232515

[ece34145-bib-0027] Hillebrand, H. , Worm, B. , & Lotze, H. (2000). Marine microbenthic community structure regulated by nitrogen loading and grazing pressure. Marine Ecology Progress Series, 204, 27–38. 10.3354/meps204027

[ece34145-bib-0028] Hiltunen, T. , Barreiro, A. , & Hairston, N. G. Jr (2012). Mixotrophy and the toxicity of Ochromonas in pelagic food webs. Freshwater Biology, 57(11), 2262–2271. 10.1111/fwb.12000

[ece34145-bib-0029] Holling, C. S. (1959). The components of predation as revealed by a study of small‐mammal predation of the European pine sawfly. The Canadian Entomologist, 91(05), 293–320. 10.4039/Ent91293-5

[ece34145-bib-0030] Holt, R. D. (1977). Predation, apparent competition, and the structure of prey communities. Theoretical Population Biology, 12(2), 197–229. 10.1016/0040-5809(77)90042-9 929457

[ece34145-bib-0031] Jones, L. E. , & Ellner, S. P. (2007). Effects of rapid prey evolution on predator–prey cycles. Journal of Mathematical Biology, 55(4), 541–573. 10.1007/s00285-007-0094-6 17483952

[ece34145-bib-0032] Jones, E. , Oliphant, T. , & Peterson, P. (2001). SciPy: Open source scientific tools for Python, 2009.

[ece34145-bib-0033] Kasada, M. , Yamamichi, M. , & Yoshida, T. (2014). Form of an evolutionary tradeoff affects eco‐evolutionary dynamics in a predator–prey system. Proceedings of the National Academy of Sciences of the United States of America, 111(45), 16035–16040. 10.1073/pnas.1406357111 25336757 PMC4234545

[ece34145-bib-0034] Kretzschmar, M. , Nisbet, R. , & Mccauley, E. (1993). A predator‐prey model for zooplankton grazing on competing algal populations. Theoretical Population Biology, 44(1), 32–66. 10.1006/tpbi.1993.1017

[ece34145-bib-0035] Leibold, M. A. (1996). A graphical model of keystone predators in food webs: Trophic regulation of abundance, incidence, and diversity patterns in communities. The American Naturalist, 147(5), 784–812. 10.1086/285879

[ece34145-bib-0036] Lima, S. L. , & Dill, L. M. (1990). Behavioral decisions made under the risk of predation: A review and prospectus. Canadian Journal of Zoology, 68(4), 619–640. 10.1139/z90-092

[ece34145-bib-0037] Lind, E. M. , Borer, E. , Seabloom, E. , Adler, P. , Bakker, J. D. , Blumenthal, D. M. , … Wragg, P. D. (2013). Life‐history constraints in grassland plant species: A growth‐defence trade‐off is the norm. Ecology Letters, 16(4), 513–521. 10.1111/ele.12078 23347060

[ece34145-bib-0038] Litchman, E. , Edwards, K. F. , & Klausmeier, C. A. (2015). Microbial resource utilization traits and trade‐offs: Implications for community structure, functioning, and biogeochemical impacts at present and in the future. Frontiers in Microbiology, 6, 254. 25904900 10.3389/fmicb.2015.00254 PMC4389539

[ece34145-bib-0039] McCann, K. S. (2011). Food webs (MPB‐50), volume 50.

[ece34145-bib-0040] McCann, K. , Hastings, A. , & Huxel, G. R. (1998). Weak trophic interactions and the balance of nature. Nature, 395(6704), 794–798. 10.1038/27427

[ece34145-bib-0041] McPeek, M. A. (1998). The consquences of changing the top predator in a foodweb: A comparative experimental approach. Ecological Monographs, 68(1), 1.

[ece34145-bib-0042] Menge, B. A. , Berlow, E. L. , Blanchette, C. A. , Navarrete, S. A. , & Yamada, S. B. (1994). The keystone species concept: Variation in interaction strength in a rocky intertidal habitat. Ecological Monographs, 64(3), 249–286. 10.2307/2937163

[ece34145-bib-0043] Meyer, J. R. , Ellner, S. P. , Hairston, N. G. , Jones, L. E. , & Yoshida, T. (2006). Prey evolution on the time scale of predator‐prey dynamics revealed by allele‐specific quantitative PCR. Proceedings of the National Academy of Sciences of the United States of America, 103(28), 10690–10695. 10.1073/pnas.0600434103 16807296 PMC1502293

[ece34145-bib-0044] Monod, J. (1950). La technique de culture continue: Theorie et applications. Annals de l'Institut Pasteur, 79, 390–410.

[ece34145-bib-0045] Murdoch, W. W. (1969). Switching in general predators: Experiments on predator specificity and stability of prey populations. Ecological Monographs, 39(4), 335–354. 10.2307/1942352

[ece34145-bib-0046] Paine, R. T. (1966). Food web complexity and species diversity. The American Naturalist, 100(910), 65–75. 10.1086/282400

[ece34145-bib-0047] Peter, H. , & Lampert, W. (1989). The effect of *Daphnia* body size on filtering rate inhibition in the presence of a filamentous cyanobacterium. Limnology and Oceanography, 34(6), 1084–1089. 10.4319/lo.1989.34.6.1084

[ece34145-bib-0048] Porter, K. G. (1973). Selective grazing and differential digestion of algae by zooplankton. Nature, 244(5412), 179–180. 10.1038/244179a0

[ece34145-bib-0049] Proulx, M. , & Mazumder, A. (1998). Reversal of grazing impact on plant species richness in nutrient‐poor vs. nutrient‐rich ecosystems. Ecology, 79(8), 2581–2592. 10.1890/0012-9658(1998)079[2581:ROGIOP]2.0.CO;2

[ece34145-bib-0050] Raatz, M. , Gaedke, U. , & Wacker, A. (2017). High food quality of prey lowers its risk of extinction. Oikos, 126(10), 1501–1510. 10.1111/oik.03863

[ece34145-bib-0051] Rueffler, C. , Van Dooren, T. J. M. , & Metz, J. A. J. (2006). The evolution of resource specialization through frequency‐dependent and frequency‐independent mechanisms. The American Naturalist, 167(1), 81–93. 10.1086/498275 16475101

[ece34145-bib-0052] Siemens, D. H. , Garner, S. H. , Mitchell‐Olds, T. , & Callaway, R. M. (2002). Cost of defense in the context of plant competition: Brassica rapa may grow and defend. Ecology, 83(2), 505–517. 10.1890/0012-9658(2002)083[0505:CODITC]2.0.CO;2

[ece34145-bib-0053] Sih, A. , Crowley, P. , McPeek, M. , Petranka, J. , & Strohmeier, K. (1985). Predation, competition, and prey communities: A review of field experiments. Annual Review of Ecology and Systematics, 16(1), 269–311. 10.1146/annurev.es.16.110185.001413

[ece34145-bib-0054] Smith, S. L. , Merico, A. , Wirtz, K. W. , & Pahlow, M. (2014). Leaving misleading legacies behind in plankton ecosystem modelling. Journal of Plankton Research, 36(3), 613–620. 10.1093/plankt/fbu011

[ece34145-bib-0055] Smith, H. L. , & Waltman, P. (1995). The theory of the chemostat: Dynamics of microbial competition. Cambridge studies in mathematical biology. Cambridge, UK: Cambridge University Press 10.1017/CBO9780511530043

[ece34145-bib-0056] Stearns, S. C. (1989). Trade‐offs in life‐history evolution. Functional Ecology, 3(3), 259 10.2307/2389364

[ece34145-bib-0057] Stevens, M. , & Ruxton, G. D. (2012). Linking the evolution and form of warning coloration in nature. Proceedings of the Royal Society B: Biological Sciences, 279(1728), 417–426. 10.1098/rspb.2011.1932 22113031 PMC3234570

[ece34145-bib-0058] Strauss, S. Y. , Rudgers, J. A. , Lau, J. A. , & Irwin, R. E. (2002). Direct and ecological costs of resistance to herbivory. Trends in Ecology and Evolution, 17(6), 278–285. 10.1016/S0169-5347(02)02483-7

[ece34145-bib-0059] Thingstad, T. F. (2000). Elements of a theory for the mechanisms controlling abundance, diversity, and biogeochemical role of lytic bacterial viruses in aquatic systems. Limnology and Oceanography, 45(6), 1320–1328. 10.4319/lo.2000.45.6.1320

[ece34145-bib-0060] Tilman, D. (1982). Resource competition and community structure. Princeton, NJ: Princeton University Press. 7162524

[ece34145-bib-0061] Tirok, K. , & Gaedke, U. (2010). Internally driven alternation of functional traits in a multispecies predator–prey system. Ecology, 91(6), 1748–1762. 10.1890/09-1052.1 20583716

[ece34145-bib-0062] van Leeuwen, C. H. A. , van der Velde, G. , van Lith, B. , & Klaassen, M. (2012). Experimental quantification of long distance dispersal potential of aquatic snails in the gut of migratory birds. PLoS One, 7(3), e32292 10.1371/journal.pone.0032292 22403642 PMC3293790

[ece34145-bib-0063] van Velzen, E. , & Etienne, R. S. (2015). The importance of ecological costs for the evolution of plant defense against herbivory. Journal of Theoretical Biology, 372, 89–99. 10.1016/j.jtbi.2015.02.027 25747775

[ece34145-bib-0064] Verdolin, J. L. (2006). Meta‐analysis of foraging and predation risk trade‐offs in terrestrial systems. Behavioral Ecology and Sociobiology, 60(4), 457–464. 10.1007/s00265-006-0172-6

[ece34145-bib-0065] Viola, D. V. , Mordecai, E. A. , Jaramillo, A. G. , Sistla, S. A. , Albertson, L. K. , Gosnell, J. S. , … Levine, J. M. (2010). Competition‐defense tradeoffs and the maintenance of plant diversity. Proceedings of the National Academy of Sciences of the United States of America, 107(40), 17217–17222. 10.1073/pnas.1007745107 20855605 PMC2951440

[ece34145-bib-0066] Vos, M. , Berrocal, S. M. , Karamaouna, F. , Hemerik, L. , & Vet, L. E. M. (2001). Plant‐mediated indirect effects and the persistence of parasitoid‐herbivore communities. Ecology Letters, 4, 38–45. 10.1046/j.1461-0248.2001.00191.x

[ece34145-bib-0067] Wada, S. , Kawakami, K. , & Chiba, S. (2012). Snails can survive passage through a bird's digestive system. Journal of Biogeography, 39(1), 69–73. 10.1111/j.1365-2699.2011.02559.x

[ece34145-bib-0068] White, J. D. , Kaul, R. B. , Knoll, L. B. , Wilson, A. E. , & Sarnelle, O. (2011). Large variation in vulnerability to grazing within a population of the colonial phytoplankter, *Microcystis aeruginosa* . Limnology and Oceanography, 56(5), 1714–1724. 10.4319/lo.2011.56.5.1714

[ece34145-bib-0069] Winter, C. , Bouvier, T. , Weinbauer, M. G. , & Thingstad, T. F. (2010). Trade‐offs between competition and defense specialists among unicellular planktonic organisms: The “killing the winner” hypothesis revisited. Microbiology and Molecular Biology Reviews, 74(1), 42–57. 10.1128/MMBR.00034-09 20197498 PMC2832346

[ece34145-bib-0070] Xu, J. , Nielsen, L. T. , & Kiørboe, T. (2018). Foraging response and acclimation of ambush feeding and feeding‐current feeding copepods to toxic dinoflagellates. Limnology and Oceanography, in press, 10.1002/lno.10782

[ece34145-bib-0071] Yamauchi, A. , & Yamamura, N. (2005). Effects of defense evolution and diet choice on population dynamics in a one‐predator‐two‐prey system. Ecology, 86(9), 2513–2524. 10.1890/04-1524

[ece34145-bib-0072] Yoshida, T. , Ellner, S. P. , Jones, L. E. , Bohannan, B. J. M. , Lenski, R. E. , & Hairston, N. G. Jr (2007). Cryptic population dynamics: Rapid evolution masks trophic interactions. PLoS Biology, 5(9), e235 10.1371/journal.pbio.0050235 17803356 PMC1964773

[ece34145-bib-0073] Yoshida, T. , Hairston, N. G. , & Ellner, S. P. (2004). Evolutionary trade‐off between defence against grazing and competitive ability in a simple unicellular alga, *Chlorella vulgaris* . Proceedings of the Royal Society B: Biological Sciences, 271(1551), 1947–1953. 10.1098/rspb.2004.2818 15347519 PMC1691804

[ece34145-bib-0074] Yoshida, T. , Jones, L. E. , Ellner, S. P. , Fussmann, G. F. , & Hairston, N. G. (2003). Rapid evolution drives ecological dynamics in a predator‐prey system. Nature, 424(July), 303–306. 10.1038/nature01767 12867979

