# Supplementary material for: Not attackable or not crackable—How pre‐ and post‐attack defenses with different competition costs affect prey coexistence and population dynamics
Source: Ecol Evol. 2018 Jun 11;8(13):6625–37. doi: 10.1002/ece3.4145 (PMC6053555; doi:10.1002/ece3.4145)
Supplement: Supplementary file 1 [file ECE3-8-6625-s001.pdf]

Not attackable or not crackable - How pre- and post-attack defenses with different competition costs affect prey coexistence and population dynamics

Elias Ehrlich and Ursula Gaedke

Ecology and Evolution, 2018

Appendix S1: Digestion resistance

Species may defend at different points of the predation sequence (sequence of encounter, attacking, capturing, manipulating, ingestion, digestion). In the main text, we distinguished between defenses preventing attacks (low attack probability p_i) and those operating subsequent to attacks by reducing the consumption probability q_i. Empirical studies revealed that certain species may defend themselves even after being ingested by surviving the gut passage, e.g., aquatic snails eaten by birds (van Leeuwen et al. 2012; Wada et al. 2012) or phytoplankton species with thickened cell walls consumed by different zooplankton species (Porter 1973; Meyer et al. 2006; Demott and McKinney 2015). To implement such a digestion resistance into the functional response of the predator, we introduce the digestion probability d_i of the prey. We define a prey only as digestion resistant if it passes the digestive system of the predator alive. The probability of surviving the gut passage after being consumed is given by $1-d_i$. Low-quality prey, which is killed by consumption but mainly excreted afterwards, is not regarded as defended/ digestion resistant. Such food-quality effects would be implemented by a low conversion efficiency of the predator rather than a low d_i (Raatz et al. 2017).

We consider a Holling type II functional response F_i of the predator for the prey A_i. It should be mentioned that, by including the digestion probability d_i into the functional response, F_i represents the rate of prey digestion instead of prey consumption

$$F_i = \frac{a\,p_i\,q_i\,d_i A_i}{1 + ap_1(c_aT + q_1(1-c_a)T)A_1 + ap_2(c_aT + q_2(1-c_a)T)A_2}\,. \tag{A1}$$

A predator invests even more time in handling a digestion resistant A_1 compared to a post-attack defended A_1 (see Tab. 1 in the main text), i.e., not only the attack time T_a (c_aT) but also the manipulation time T_m ($(1-c_a)T$). Thus, the indirect facilitation of A_2 by A_1 increases and the coexistence region is enlarged in comparison to a post-attack defense (Fig. 2 and A1). The effects of digestion resistance and post-attack defenses are equal if the main part of the handling time is needed for

attacking ($c_a \approx 1$).

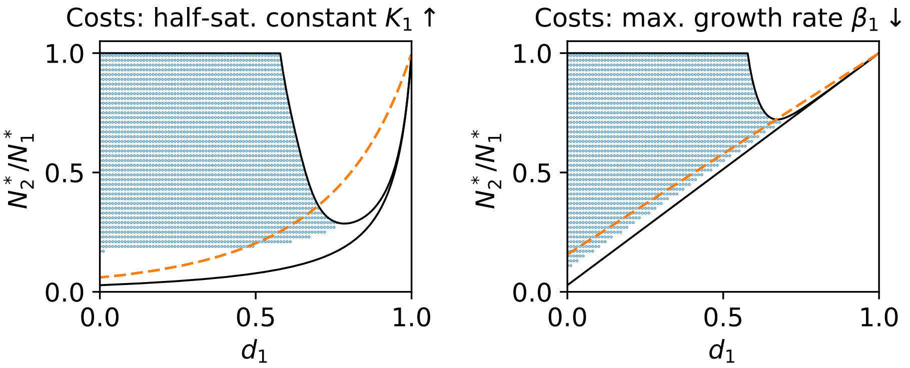

Figure A1: Coexistence and population dynamics of a digestion resistant prey A_1 and an undefended prey A_2 in dependence of the digestion probability d_1 of A_1 and its half-saturation constant for resource uptake K_1 or its maximum growth rate β_1. The trait values of A_2 are kept constant. The y-axis represents the relative competitiveness N_2^*/N_1^* of A_1 compared to A_2. The resource supply is set to N_I=160 µmol N/l. The black lines enclose the region where a coexistence equilibrium exists and blue dots mark where it is locally stable. The dashed orange line represents the invasion boundary of A_1 invading a resident community with A_2 (invasion is possible above the line).

References

Demott, W. R. and McKinney, E. N. (2015). Use it or lose it? Loss of grazing defenses during laboratory culture of the digestion-resistant green alga Oocystis. *Journal of Plankton Research*, 37(2):399–408.

Meyer, J. R., Ellner, S. P., Hairston, N. G., Jones, L. E., and Yoshida, T. (2006). Prey evolution on the time scale of predator-prey dynamics revealed by allele-specific quantitative PCR. *Proceedings of the National Academy of Sciences of the United States of America*, 103(28):10690–10695.

Porter, K. G. (1973). Selective Grazing and Differential Digestion of Algae by Zooplankton. *Nature*, 244(5412):179–180.

Raatz, M., Gaedke, U., and Wacker, A. (2017). High food quality of prey lowers its risk of extinction. *Oikos*, 126(10):1501–1510.

van Leeuwen, C. H. A., van der Velde, G., van Lith, B., and Klaassen, M. (2012). Experimental Quantification of Long Distance Dispersal Potential of Aquatic Snails in the Gut of Migratory Birds. *PLoS ONE*, 7(3):e32292.

Wada, S., Kawakami, K., and Chiba, S. (2012). Snails can survive passage through a bird's digestive system. *Journal of Biogeography*, 39(1):69–73.
