# Supplementary material for: Not attackable or not crackable—How pre‐ and post‐attack defenses with different competition costs affect prey coexistence and population dynamics
Source: Ecol Evol. 2018 Jun 11;8(13):6625–37. doi: 10.1002/ece3.4145 (PMC6053555; doi:10.1002/ece3.4145)
Supplement: Supplementary file 2 [file ECE3-8-6625-s002.pdf]

Not attackable or not crackable - How pre- and post-attack defenses with different competition costs affect prey coexistence and population dynamics

Elias Ehrlich and Ursula Gaedke

Ecology and Evolution, 2018

Appendix S2: Coexistence equilibria, linear stability analysis and invasion boundary

The calculation of coexistence equilibria, the linear stability analysis and the calculation of the invasion boundary are based on Jones and Ellner (2007). These calculations demand a rescaling of the model. At the end of the calculations, the traits are transferred back into their original units (prior to rescaling) to present the results in the main text.

Rescaling and reducing the dimensions of the predator-prey model

The analysed predator-prey model is given by

$$\begin{aligned}
\frac{dN}{dt} &= \delta\,(N_I - N) - \sum_{i=1}^{2} \frac{\beta_i}{\chi}\frac{N A_i}{K_i + N} \\
\frac{dA_i}{dt} &= A_i\left[\beta_i \frac{N}{K_i+N} - \frac{a\,p_i\,q_i\,d_i\,P}{1 + a\sum p_i(c_a T + q_i(1-c_a)T)A_i} - \delta\right] \\
\frac{dP}{dt} &= P\left[\chi_P \frac{a\sum p_i\,q_i\,d_i A_i}{1 + a\sum p_i(c_a T + q_i(1-c_a)T)A_i} - \delta\right]
\end{aligned} \tag{B1}$$

with $i = 1,2$. In addition to the model presented in the main text (Eq. 2), we included also the digestion probability d_i (see Appendix A). To allow for analytical calculations, we rescale the model with the following substitutions

$$\begin{aligned}
&\tau = \delta\,t,\; S = \frac{N}{N_I},\; x_i = \frac{A_i}{\chi N_I},\; y = \frac{P}{\chi_P\,\chi\,N_I}, \\
&g = \frac{\chi_P}{\delta\,T},\; k_P = \frac{1}{\chi\,N_I\,a\,T},\; k_i = \frac{K_i}{N_I},\; r_i = \frac{\beta_i}{\delta}\,.
\end{aligned} \tag{B2}$$

The rescaling ensures that all variables and parameters are dimensionless. The following equations represent the rescaled model

$$\begin{aligned}
\dot{S} &= 1 - S - \sum_{i=1}^{2} \frac{r_i S x_i}{k_i + S} \\
\dot{x}_i &= x_i \left[\frac{r_i S}{k_i + S} - \frac{g\, p_i q_i d_i y}{k_P + \sum p_i \left(c_a + q_i(1 - c_a)\right) x_i} - 1 \right] \\
\dot{y} &= y \left[\frac{g \sum p_i q_i d_i x_i}{k_P + \sum p_i \left(c_a + q_i(1 - c_a)\right) x_i} - 1 \right] .
\end{aligned} \tag{B3}$$

The derivatives over time t ($\frac{dN}{dt}$, $\frac{dA_i}{dt}$, $\frac{dB}{dt}$) are transformed into derivatives over τ ($\dot{S}$, $\dot{x}_i$ and $\dot{y}$). The sum of the population densities $\sum = S + x_1 + x_2 + y$ is changing over time in dependence of the rate of change of its components

$$\dot{\sum} = \dot{S} + \dot{x}_1 + \dot{x}_2 + \dot{y} \tag{B4}$$

Inserting the Eq. B3 into Eq. B4 results in

$$\begin{aligned}
\dot{\sum} &= 1 - S - x_1 - x_2 - y \\
\dot{\sum} &= 1 - \sum
\end{aligned} \tag{B5}$$

$\dot{\sum}$ becomes zero when $\sum$ gets close to one implying that $\sum = 1$ is an equilibrium (Jones and Ellner 2007). Thus, in the long-term, $\sum$ has a constant value of one meaning that the joint capacity of the populations S, x_1, x_2 and y in the chemostat system is one. Considering long-term dynamics allows the following substitution for the resource density by inserting $\sum = 1$ into $\sum = S + x_1 + x_2 + y$ and rearranging this equation

$$S = 1 - x_1 - x_2 - y\,. \tag{B6}$$

Thus, the resource density can be represented by a function of the densities of the prey types and the predator (Eq. B6) allowing for a dimensional reduction of the model (Jones and Ellner 2007). The rescaled, reduced model is represented by the following equations

$$\begin{aligned}
\dot{x}_i &= x_i \left[\frac{r_i (1 - x_1 - x_2 - y)}{k_i + (1 - x_1 - x_2 - y)} - \frac{g\, p_i q_i d_i y}{k_P + \sum p_i \left(c_a + q_i(1 - c_a)\right) x_i} - 1 \right] \\
\dot{y} &= y \left[\frac{g \sum p_i q_i d_i x_i}{k_P + \sum p_i \left(c_a + q_i(1 - c_a)\right) x_i} - 1 \right] .
\end{aligned} \tag{B7}$$

Coexistence equilibrium

One goal of this study is to find the conditions for coexistence of both prey types. Without a loss of generality, we explain our method based on the attack probability-maximum growth rate trade-off (p-β-TO) where the prey types do not differ in their consumption probability, digestion probability and their half-saturation constant, i.e., we assume that $q_1 = q_2 = 1$, $d_1 = d_2 = 1$ and $k_1 = k_2 = k$. One condition for coexistence is the existence of an equilibrium ($\dot{x}_1$, $\dot{x}_2$ and $\dot{y}$ equal to zero) where all population densities are positive. This kind of equilibrium is called coexistence equilibrium (Jones and Ellner 2007). Equilibrium population densities are marked with a tilde. By inserting $\dot{y} = 0$ into Eq. B7 the expression for the total attackable prey density at equilibrium ($\widetilde{Q} = \sum p_i \widetilde{x_i}$) is derived as

$$\widetilde{Q} = \frac{k_P}{g-1} \Leftrightarrow \frac{g}{k_P + \widetilde{Q}} = \frac{1}{\widetilde{Q}} \tag{B8}$$

The substitution $S = 1 - x_1 - x_2 - y$ simplifies the further analysis (Eq. B6). The solution for the equilibrium predator density for $\dot{x}_1 = 0$ is

$$\widetilde{y} = \frac{\widetilde{Q}}{p_1} \left[\frac{(r_1 - 1)\widetilde{S} - k}{k + \widetilde{S}} \right] \tag{B9}$$

and for $\dot{x}_2 = 0$

$$\widetilde{y} = \frac{\widetilde{Q}}{p_2} \left[\frac{(r_2 - 1)\widetilde{S} - k}{k + \widetilde{S}} \right] \tag{B10}$$

These solutions (Eq. B9 and B10) have to be equal. Equating both terms results in

$$\widetilde{S} = \frac{(1 - \frac{p_1}{p_2})k}{\frac{p_1}{p_2} - \frac{p_1}{p_2} r_2 + r_1 - 1} \tag{B11}$$

$\widetilde{S}$ corresponds to the equilibrium resource density. The total prey density in equilibrium $\widetilde{X}$ ($= \widetilde{x_1} + \widetilde{x_2}$) is obtained by inserting $\widetilde{S}$ (Eq. B11) and $\widetilde{y}$ (Eq. B9 or B10) into $\widetilde{X} = 1 - \widetilde{S} - \widetilde{y}$. As explained in Jones and Ellner (2007), the equilibrium prey population densities $\widetilde{x_1}$ and $\widetilde{x_2}$ can be represented as

$$\begin{bmatrix} \widetilde{x_1} \\ \widetilde{x_2} \end{bmatrix} = \frac{1}{p_2 - p_1} \begin{bmatrix} p_2 \widetilde{X} - \widetilde{Q} \\ \widetilde{Q} - p_1 \widetilde{X} \end{bmatrix} \tag{B12}$$

Due to the coexistence condition stating that equilibrium population densities have to be positive ($\widetilde{x_1}$ and $\widetilde{x_2} > 0$) and remembering that $p_1 < p_2$ it follows from Equation B12 that $p_1 \widetilde{X} < \widetilde{Q} < p_2 \widetilde{X}$

(Jones and Ellner 2007). This term can be rearranged to

$$p_1 < \frac{\widetilde{Q}}{\widetilde{\widetilde{X}}} < p_2 \tag{B13}$$

The Inequation B13 defines the condition for the coexistence equilibrium where $\frac{\widetilde{Q}}{\widetilde{\widetilde{X}}}$ can be considered as a density-weighted mean attack probability of the prey types. The attack probability of the defended prey is smaller and the attack probability of the undefended prey is larger than this density-weighed mean attack probability when there is a coexistence equilibrium.

Linear stability analysis

Another goal of this study is to identify the dynamics potentially occurring in this predator-prey system. A linear stability analysis enables to distinguish between locally stable equilibria (i.e. steady-state) and locally unstable equilibria (e.g. existence of stable limit cycles). Therefore, we analyse the local stability of the coexistence equilibrium. We refer here exemplary again to the p-β-TO and assume that $d_1 = d_2 = 1$ and $k_1 = k_2 = k$. The further steps are based on the study of Jones and Ellner (2007). The Jacobian matrix at the coexistence equilibrium J is generated by computing the necessary partial derivatives for the rescaled, reduced model (Eq. B7).

$$J = \left.\begin{pmatrix} \frac{\partial \dot{x}_1}{\partial x_1} & \frac{\partial \dot{x}_1}{\partial x_2} & \frac{\partial \dot{x}_1}{\partial y} \\ \frac{\partial \dot{x}_2}{\partial x_1} & \frac{\partial \dot{x}_2}{\partial x_2} & \frac{\partial \dot{x}_2}{\partial y} \\ \frac{\partial \dot{y}}{\partial x_1} & \frac{\partial \dot{y}}{\partial x_2} & \frac{\partial \dot{y}}{\partial y} \end{pmatrix}\right|_{x_1=\widetilde{x_1}, x_2=\widetilde{x_2}, y=\widetilde{y}}$$

$$= \begin{pmatrix} \widetilde{x_1}\left[u_{x_1} - p_1^2 \widetilde{y}\widetilde{v}'\right] & \widetilde{x_1}\left[u_{x_1} - p_1 p_2 \widetilde{y}\widetilde{v}'\right] & \widetilde{x_1}\left[u_{y_1} - p_1 \widetilde{v}\right] \\ \widetilde{x_2}\left[u_{x_2} - p_1 p_2 \widetilde{y}\widetilde{v}'\right] & \widetilde{x_2}\left[u_{x_2} - p_2^2 \widetilde{y}\widetilde{v}'\right] & \widetilde{x_2}\left[u_{y_2} - p_2 \widetilde{v}\right] \\ p_1 \widetilde{y}\widetilde{w}' & p_2 \widetilde{y}\widetilde{w}' & 0 \end{pmatrix} \tag{B14}$$

$$= \begin{pmatrix} a & b & c \\ d & e & f \\ g & h & i \end{pmatrix}$$

with

$$
\begin{aligned}
u_{x_1} = u_{y_1} &= -\frac{r_1 k}{(k+1-\widetilde{X}-\widetilde{y})^2} \\
u_{x_2} = u_{y_2} &= -\frac{r_2 k}{(k+1-\widetilde{X}-\widetilde{y})^2} \\
\widetilde{v} &= \frac{g}{k_P+\widetilde{Q}} \\
\widetilde{v}' &= -\frac{g}{(k_P+\widetilde{Q})^2} \\
\widetilde{w}' &= -\frac{g\,k_P}{(k_P+\widetilde{Q})^2}
\end{aligned}
\tag{B15}
$$

The roots of the characteristic polynomial of J are the eigenvalues λ of J. If all eigenvalues have negative real parts then the equilibrium is locally stable. The general equation of characteristic polynomials for a 3×3 dimensional matrix is

$$
p(\lambda) = \det(\lambda I - J) = \lambda^3 + c_2\lambda^2 + c_1\lambda + c_0 = 0 \tag{B16}
$$

The coefficients of the characteristic polynomial are

$$
\begin{aligned}
c_0 &= -(aei + bfg + cdh - gec - hfa - idb) = -\det(J) \\
c_1 &= ae + ai + ei - db - gc - hf \\
c_2 &= -(a+e+i) = -\mathrm{trace}(J)
\end{aligned}
\tag{B17}
$$

The small letters in the definition of the coefficients represent the elements of the Jacobian matrix shown in Equation B14. The coefficient c_0 is equal to the negative determinant of J while c_2 equals the negative trace of J (Jones and Ellner 2007). The Routh-Hurwitz stability criterion (May 1974) is used for revealing whether the coexistence equilibrium is locally stable. According to this criterion, all eigenvalues (roots of the characteristic polynomial, Eq. B16) have negative real parts when the following conditions hold

$$
\begin{aligned}
I)\; & c_0 > 0 \\
II)\; & c_1 > 0 \\
III)\; & c_2 > 0 \\
IV)\; & c_1 c_2 > c_0\,.
\end{aligned}
\tag{B18}
$$

Invasion boundary

Here we derive the invasion boundary of the defended prey A_1 invading a resident community of the undefended prey A_2 and the predator P. This demands no rescaling of the model. Hence, we refer to the original model (Eq. B1). We explain the calculation again based on the p-β-TO where $q_1 = q_2 = 1$, $d_1 = d_2 = 1$ and $K_1 = K_2 = K$. The invasion fitness of A_1 which is defined as the long-term mean per capita growth rate at very low densities ($A_1 \approx 0$) is given by

$$\left\langle \frac{1}{A_1}\frac{dA_1}{dt} \right\rangle = \beta_1 \left\langle \frac{N}{K+N} \right\rangle - p_1 \left\langle \frac{aP}{1+a\,p_2\,(c_a T + (1-c_a)T)\,A_2} \right\rangle - \delta \tag{B19}$$

where long-term means are indicated by angle brackets. At the invasion boundary, the invasion fitness equals zero, i.e.

$$0 = \beta_1 \left\langle \frac{N}{K+N} \right\rangle - p_1 \left\langle \frac{aP}{1+a\,p_2\,T\,A_2} \right\rangle - \delta\,. \tag{B20}$$

This can be rearranged to

$$\beta_1 = \frac{p_1 \left\langle \frac{aP}{1+a p_2 T A_2} \right\rangle + \delta}{\left\langle \frac{N}{K+N} \right\rangle} \tag{B21}$$

which yields the relationship between the maximum growth rate β_1 and the attack probability p_1 of the defended prey at its invasion boundary. The long-term means of the densities of the resident community N, A_2 and P (Eq. B21) have to be computed numerically over one cycle of the residents.

References

Jones, L. E. and Ellner, S. P. (2007). Effects of rapid prey evolution on predator–prey cycles. *Journal of Mathematical Biology*, 55(4):541–573.

May, R. (1974). *Stability and complexity in model ecosystems*. Princeton University Press.
