# Supplementary material for: Not attackable or not crackable—How pre‐ and post‐attack defenses with different competition costs affect prey coexistence and population dynamics
Source: Ecol Evol. 2018 Jun 11;8(13):6625–37. doi: 10.1002/ece3.4145 (PMC6053555; doi:10.1002/ece3.4145)
Supplement: Supplementary file 3 [file ECE3-8-6625-s003.pdf]

Not attackable or not crackable - How pre- and post-attack defenses with different competition costs affect prey coexistence and population dynamics

Elias Ehrlich and Ursula Gaedke

Ecology and Evolution, 2018

Appendix S3: Sensitivity analysis

Here, we examine the sensitivity of the results to altered parameter values. We show that the general pattern, that post-attack defenses promote coexistence and stable dynamics more strongly than pre-attack defenses and that a higher half-saturation K_1 enhances the occurrence of coexistence and destabilizes the dynamics compared to a lower maximum growth rate β_1, is largely independent from distinct parameter values (Fig. C1-C7).

First, we test how the results depend on the resource supply N_I (Fig. C1, C2). For a low level of resource supply ($N_I = 80$ µmol N/L), differences between the two defense mechanisms in coexistence patterns are low, but significant for different defense costs (Fig. C1). The population dynamics are overall stable for such a low resource supply independent of the defense and its costs (Fig. C1). Increasing the resource supply to $N_I = 240$ µmol N/L reveals large differences in coexistence patterns and population dynamics for the different types of defenses and costs (Fig. C2).

Second, we consider the fraction of the predator handling time spent for attacking the prey c_a. With increasing values of c_a, a post-attacked defended prey demands higher handling times while a pre-attack defended prey is not handled by the predator. Thus, the patterns of coexistence and population dynamics for pre- and post-attack defenses diverge when c_a increases (Fig. C3). The results for a pre-attack defense are independent of c_a (Fig. C4). In contrast, in case of a post-attack defense, a higher c_a steadily enhances coexistence and promotes the occurrence of stable dynamics up to a value around 0.35. Above this values, the fraction of locally stable coexistence equilibria does not increased any more (Fig. C4).

For the other parameters, a recurring pattern can be observed which is generally independent from the type of defense and costs. With increasing values of the encounter rate a, the conversion efficiency of the predator χ_P, the conversion efficiency of the prey χ and the resource supply N_I, the occurrence of coexistence equilibria initially increases but declines above a certain value

while the fraction of locally stable coexistence equilibria which initially equals one is continuously decreasing (Fig. C5). Decreasing values of the total handling time T and the dilution rate δ initially increase the occurrence of coexistence equilibria but then decrease it below a certain value. The fraction of locally stable coexistence equilibria initially decreases with lower values of T and δ but then starts to increase again especially for lower values of T.

Independent of the defense mechanism and the cost type, a higher maximum growth β_2 of the undefended prey slightly enhances coexistence and promotes steady-state dynamics (Fig. C6). A similar trend can be observed for a lower half-saturation constant K_2 of the undefended prey in case of post-attack defenses while it is slightly the opposite trend for pre-attack defenses (Fig. C7).

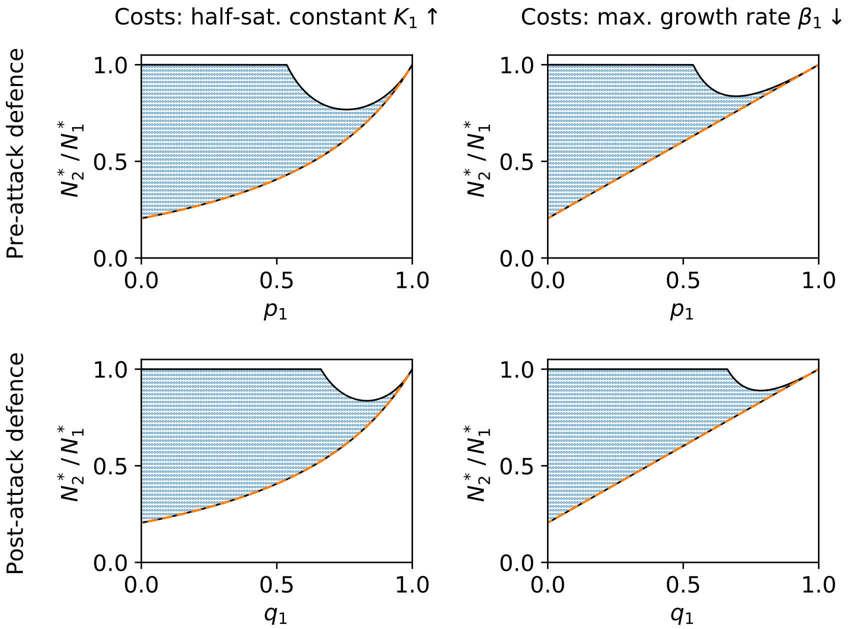

Figure C1: Low resource supply, $N_I = 80$ µmol N/l. Coexistence equilibria of the defended prey A_1 and the undefended prey A_2 in dependence of the defense mechanism (top: attack probability $p_1 < 1$, bottom: consumption probability $q_1 < 1$) and the defense costs (left: higher half-saturation constant K_1, right: lower maximum growth rate β_1). The y-axis represents the relative competitiveness of A_1 (see main text). The black lines enclose the region where a coexistence equilibrium exists and blue dots mark where it is locally stable. The dashed orange line represents the invasion boundary above which A_1 can invade a resident community with A_2.

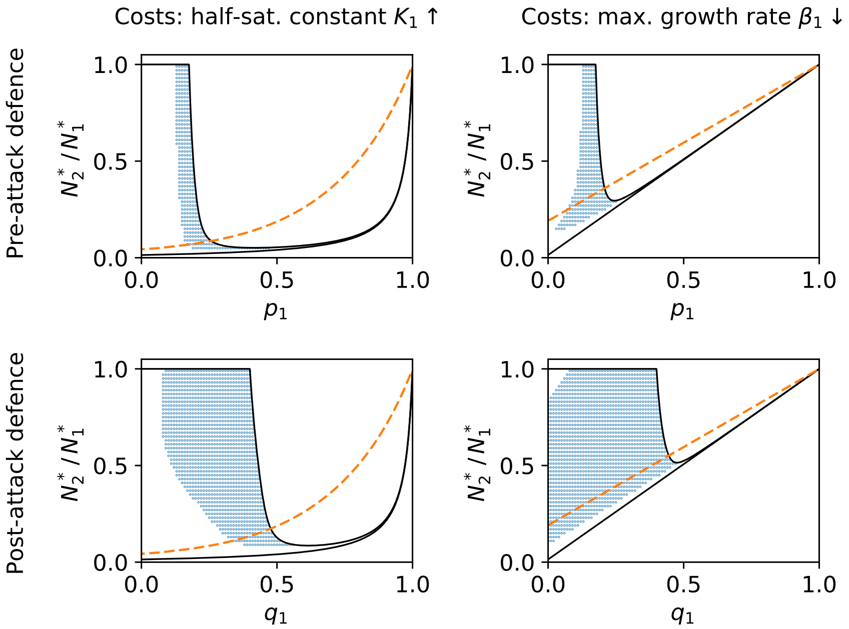

Figure C2: High resource supply, $N_I = 240$ µmol N/l. Coexistence equilibria of the defended prey A_1 and the undefended prey A_2 in dependence of the defense mechanism (top: attack probability $p_1 < 1$, bottom: consumption probability $q_1 < 1$) and the defense costs (left: higher half-saturation constant K_1, right: lower maximum growth rate β_1). The y-axis represents the relative competitiveness of A_1 (see main text). The black lines enclose the region where a coexistence equilibrium exists and blue dots mark where it is locally stable. The dashed orange line represents the invasion boundary above which A_1 can invade a resident community with A_2.

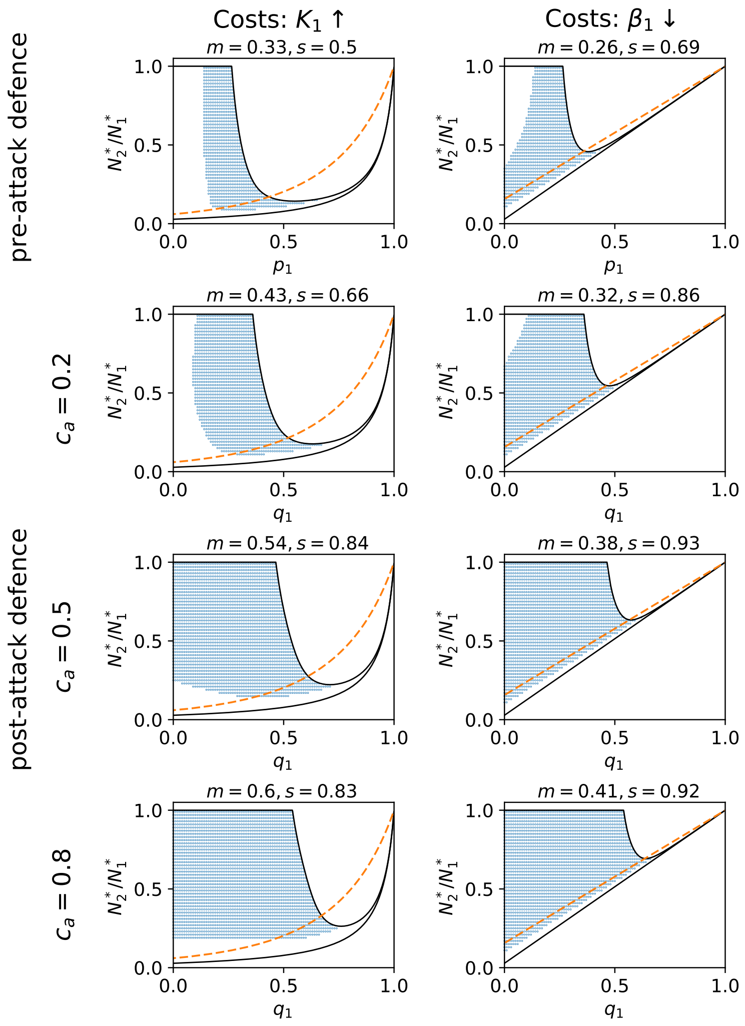

Figure C3: Coexistence equilibria of the defended prey A_1 and the undefended prey A_2 in dependence of the defense level (top panel: attack probability $p_1 < 1$, three bottom panels: consumption probability $q_1 < 1$) and the defense costs (left: higher half-saturation constant K_1, right: lower maximum growth rate β_1) for $N_I = 160$ µmol N/l. The y-axis represents the relative competitiveness of A_1 (see main text). The results for the post-attack defense depend on the fraction of the predator's handling time spent for attacking the prey c_a which increases from top to bottom. The black lines enclose the region where a coexistence equilibrium exists and blue dots mark where it is locally stable. m gives the fraction of the total trait space where a coexistence equilibrium exists while s indicates the fraction of the coexistence equilibria which are locally stable. The dashed orange line represents the invasion boundary above which A_1 can invade a resident community with A_2.

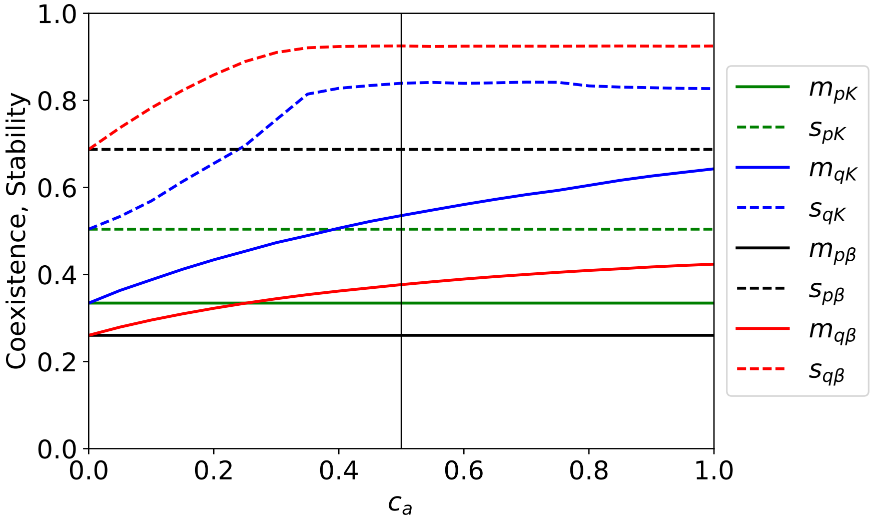

Figure C4: Coexistence equilibria and their local stability in dependence of the fraction of handling time spent for attacking the prey c_a. The vertical line marks the value of c_a used in the main text. m indicates the fraction of the total trait space where a coexistence equilibrium exists and s gives the amount of locally stable coexistence equilibria (see Fig. C3). The subscript letters refer to the considered defense trait (attack probability p or consumption probability q) and the cost trait (half-saturation constant K or maximum growth rate β).

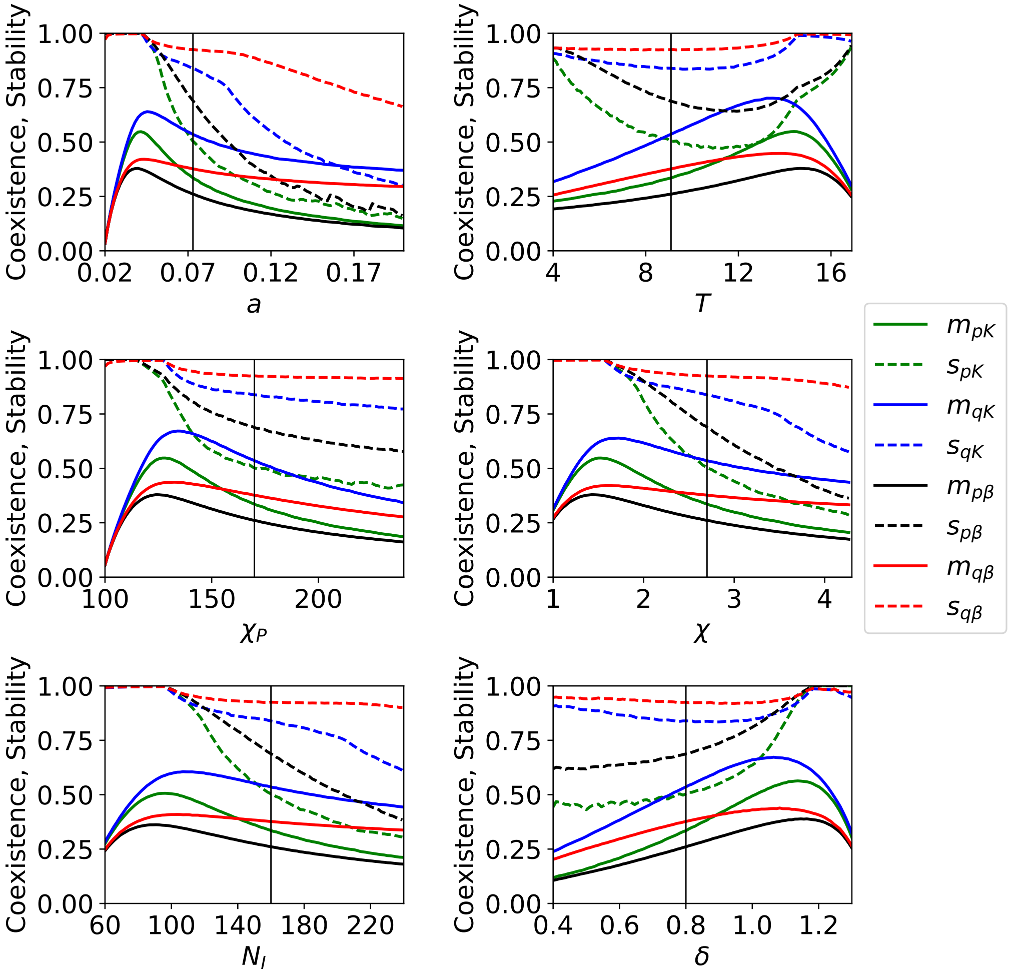

Figure C5: Coexistence equilibria and their local stability in dependence of (a) the encounter rate a (ml/d), (b) the total handling time T ($10^{-5}\,d$), (c) the predator's conversion efficiency χ_P (10^{-6}), (d) the conversion efficiency of the prey χ (10^6 ind./μmol N), (e) the resource concentration in the supplied medium N_I (μmol N/l), and (f) the dilution rate δ (d^{-1}). The vertical lines mark the parameter values used in the main text. m indicates the fraction of the total trait space where a coexistence equilibrium exists and s gives the amount of locally stable coexistence equilibria (see Fig. C3). The subscript letters refer to the considered defense trait (attack probability p or consumption probability q) and the cost trait (half-saturation constant K or maximum growth rate β).

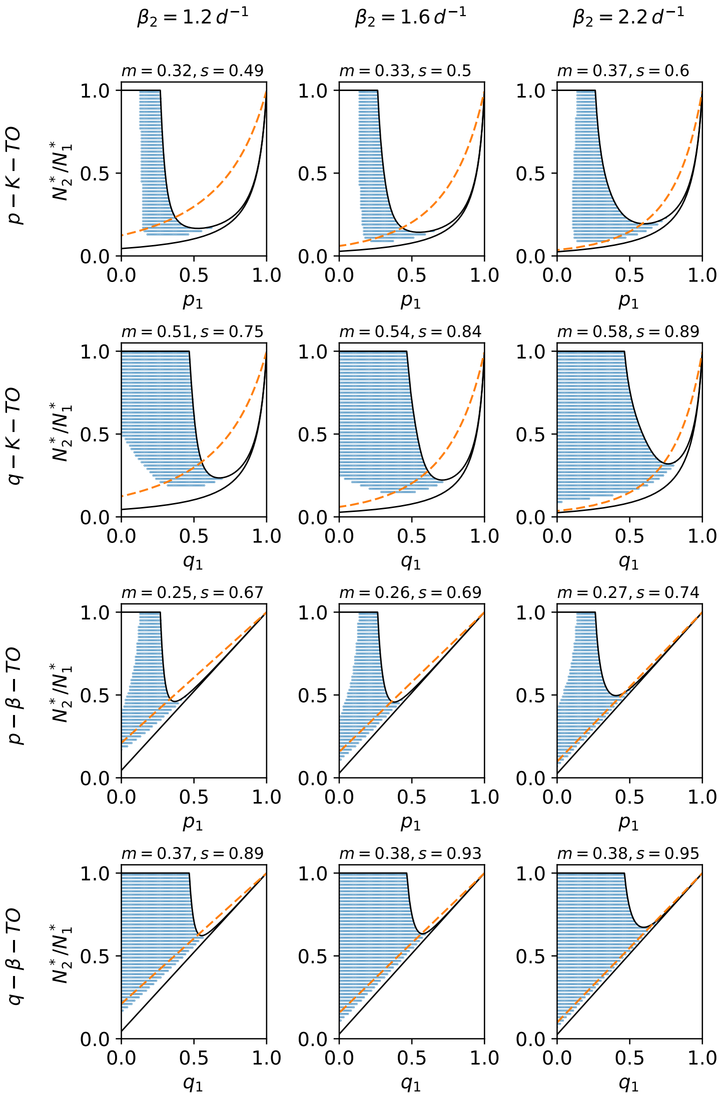

Figure C6: Coexistence equilibria of the defended prey A_1 and the undefended prey A_2 in dependence of the defense level (pre-attack defense with attack probability $p_1 < 1$ or post-attack defense with consumption probability $q_1 < 1$) and the defense costs (higher half-saturation constant K_1 or lower maximum growth rate β_1) for different maximum growth rates of the undefended prey β_2 (1.2, 1.6 or 2.2 d^{-1}). N_I is set to 160 µmol N/l. The black lines enclose the region where a coexistence equilibrium exists and blue dots mark where it is locally stable. m indicates the fraction of the total trait space where a coexistence equilibrium exists and s gives the amount of locally stable coexistence equilibria. The dashed orange line represents the invasion boundary above which A_1 can invade a resident community with A_2.

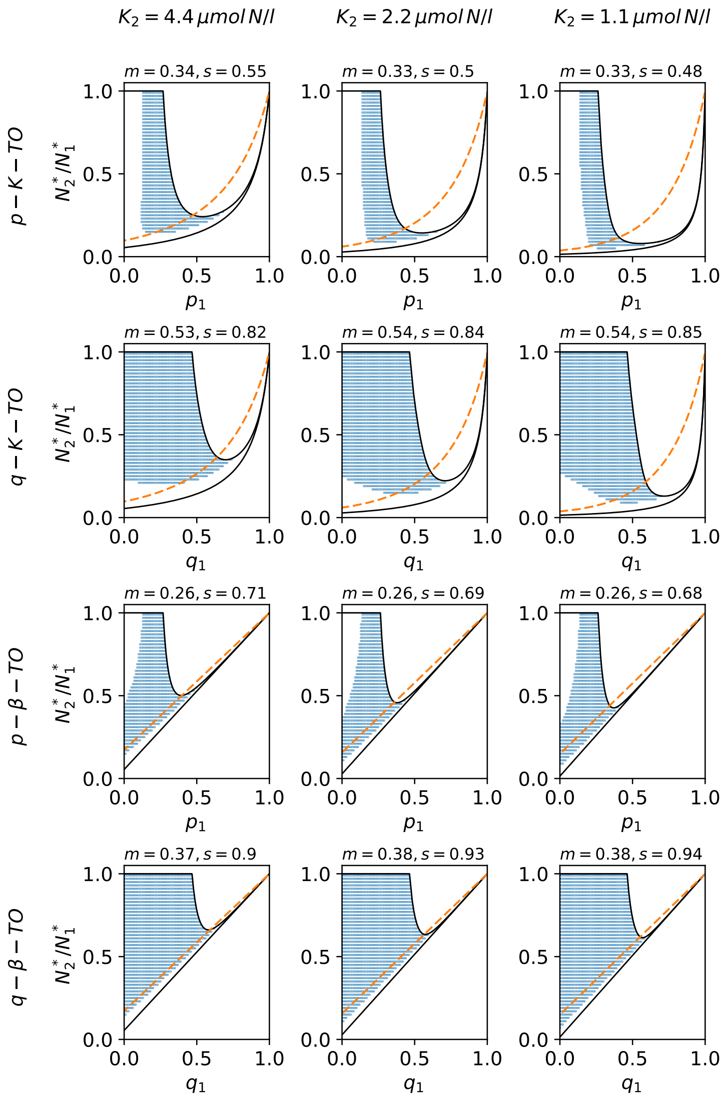

Figure C7: Coexistence equilibria of the defended prey A_1 and the undefended prey A_2 in dependence of the defense level (pre-attack defense with attack probability $p_1 < 1$ or post-attack defense with consumption probability $q_1 < 1$) and the defense costs (higher half-saturation constant K_1 or lower maximum growth rate β_1) for different values of the half-saturation constant of the undefended prey K_2 (4.4, 2.2 or 1.1 µmol N/l). N_I is set to 160 µmol N/l. The black lines enclose the region where a coexistence equilibrium exists and blue dots mark where it is locally stable. m indicates the fraction of the total trait space where a coexistence equilibrium exists and s gives the amount of locally stable coexistence equilibria. The dashed orange line represents the invasion boundary above which A_1 can invade a resident community with A_2.
