# Supplementary material for: Not attackable or not crackable—How pre‐ and post‐attack defenses with different competition costs affect prey coexistence and population dynamics
Source: Ecol Evol. 2018 Jun 11;8(13):6625–37. doi: 10.1002/ece3.4145 (PMC6053555; doi:10.1002/ece3.4145)
Supplement: Supplementary file 4 [file ECE3-8-6625-s004.pdf]

Not attackable or not crackable - How pre- and post-attack defenses with different competition costs affect prey coexistence and population dynamics

Elias Ehrlich and Ursula Gaedke

Ecology and Evolution, 2018

Appendix S4: Multistability

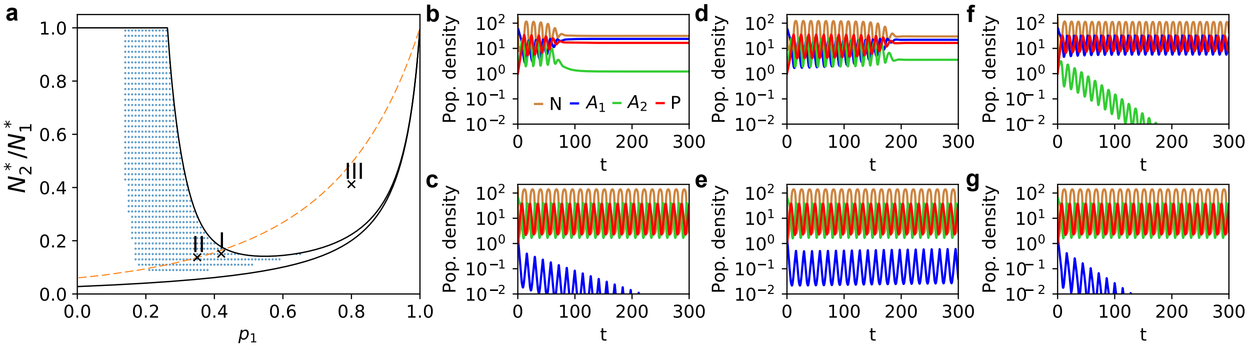

Figure D1: (a) Coexistence equilibria (within black lines), their stability (blue dots indicate local stability) and the invasion boundary of the defended prey A_1 invading a resident community with the undefended prey A_2 (dashed orange line) for a trade-off between attack probability and half-saturation constant. Multistability occurs at trait ranges where A_1 cannot invade but survives for high initial population densities. The trait combination I lies in a region where either both prey types coexist (b) or A_1 goes extinct (c) while in case of coexistence (trait combination II), the population densities are in steady state (d) or cycle (e) depending on the initial conditions. At the trait combination III either only A_1 survives (e) or is outcompeted (f). (b-g) Population dynamics with nutrients N, the two prey types A_i and the predator P. The initial densities of N (160 µmol N/l) and P (1 ind./ml) are kept constant. The initial density of A_1 is either 40×10^4 ind./ml (b, d, f) or 1×10^4 ind./ml (c, e, g) and *vice versa* for A_2.
